# Esophageal Small Cell Carcinoma: From Bench Discoveries to Bedside Therapeutics

**DOI:** 10.7150/ijbs.133104

**Published:** 2026-06-04

**Authors:** Zhe Wang, Jiayi Zhang, Cheng Ji, Jingxin Yu, Bangkun Shen, Xiangrui Meng, Zhengzheng Shan, Lulu Guan, Bingtong Yue, Dao Xin, Tongjin Zhao, Feng Wang

**Affiliations:** 1 Department of Oncology, The First Affiliated Hospital of Zhengzhou University, No. 1 Jianshe East Road, Zhengzhou, Henan 450052, China.; 2 Tianjian Laboratory for Advanced Biomedical Sciences, Zhengzhou, Henan 450052, China.; 3 State Key Laboratory of Metabolic dysregulation & the Prevention and Treatment of Esophageal Cancer, Zhengzhou, Henan 450052, China.; 4 Department of Colorectal Surgery and Oncology, Key Laboratory of Cancer Prevention and Intervention, Ministry of Education, The Second Affiliated Hospital, Zhejiang University School of Medicine, Hangzhou, Zhejiang, 310009, China.

**Keywords:** esophageal small cell carcinoma, immune microenvironment, biomarkers, therapeutic strategies

## Abstract

Small cell carcinoma of the esophagus (SCCE) is both rare and aggressive. Lacking its own guidelines, it has historically been managed with treatment models borrowed from small cell lung cancer (SCLC). However, advances in multi-omics and immunology are beginning to uncover distinct molecular and immune features in SCCE. These include frequent alterations in NOTCH1 and PTEN, ASCL1 and NEUROD1 transcriptional programs, and a highly adaptive immunosuppressive tumor microenvironment. Together, they point to unique biological traits that may be targetable. Clinically, conventional chemotherapy, radiotherapy, and surgery are being reassessed, with neoadjuvant therapy showing increasing value. Mechanism-driven strategies such as anti-angiogenic and DLL3-targeted therapies are under active exploration. At the same time, emerging biomarkers and multimodal predictive models are offering new tools for risk stratification and personalized management. Yet pathology adds further challenges: boundaries between SCCE, squamous cell or adenocarcinomas with neuroendocrine differentiation, and mixed or collision tumors remain blurred, creating diagnostic and therapeutic uncertainty. These challenges highlight an important shift—SCCE is no longer a clinical “black box” but rather a frontier that demands resolution through a translational lens. By realigning fragmented basic research with clinical evidence, this review not only presents a comprehensive picture of SCCE but also aims to provide clear therapeutic direction for this malignancy.

## 1. Introduction

Esophageal small cell carcinoma (SCCE) is a rare and highly aggressive subtype of esophageal cancer. It accounts for approximately 0.8%-2.4% of all esophageal malignancies and is characterized by rapid progression and early distant metastasis 1. Most patients are diagnosed at an advanced stage, and the prognosis remains extremely poor 2. Due to its rarity, there are no disease-specific staging or treatment guidelines. In clinical practice, management strategies are often adapted from small cell lung cancer (SCLC), but the outcomes are far from satisfactory 3-5. As a result, SCCE represents a major clinical challenge that demands greater research attention.

**Figure 1:**
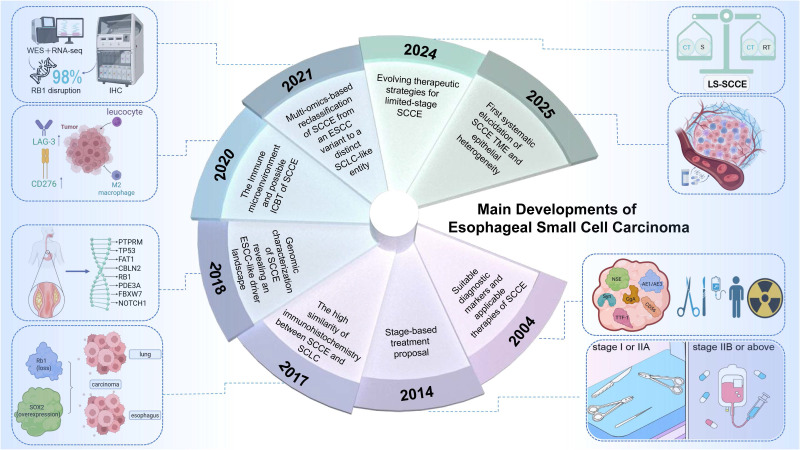
** Main developments in SCCE research.** A timeline summarizing the key milestones in the understanding of SCCE over the past two decades. Early investigations focused on its histopathologic and immunohistochemical resemblance to SCLC. Subsequent genomic studies revealed a shared driver landscape and frequent RB1 alterations. With advances in multi-omics technologies, SCCE was reclassified from a variant of esophageal squamous carcinoma to an independent SCLC-like entity. Recent work has further elucidated its tumor microenvironment, highlighting immune suppression and the potential responsiveness to immune checkpoint blockade. Meanwhile, evolving evidence has refined treatment strategies for limited-stage SCCE, moving toward stage-based and biology-informed multimodal approaches. Abbreviations: SCCE, esophageal small cell carcinoma; SCLC, small cell lung cancer; ESCC, esophageal squamous cell carcinoma; WES, whole-exome sequencing; RNA-seq, RNA sequencing; IHC, immunohistochemistry; TME, tumor microenvironment; ICBT, immune checkpoint blockade therapy; LS-SCCE, limited-stage SCCE; CT, chemotherapy; S, surgery; RT, radiotherapy; NSE, neuron-specific enolase; Syn, synaptophysin; CgA, chromogranin A; TTF-1, thyroid transcription factor 1; AE1/AE3, pan-cytokeratin.

Recent studies have begun to reveal the biological complexity of SCCE. Multi-omics approaches, including whole-exome sequencing, transcriptome profiling, and single-cell RNA sequencing, have uncovered key genetic alterations and tumor microenvironment features. Frequent disruptions in RB1 and abnormal expression of SOX2, NEUROD1, and ASCL1 suggest similarities to SCLC, but SCCE also exhibits unique molecular traits 6-8. Immunologically, SCCE is characterized by a suppressive tumor microenvironment marked by regulatory T cell (Treg) enrichment and angiogenesis-associated niches, suggesting potential resistance to immune checkpoint blockade 9, 10. However, due to limited sample sizes and the rarity of the disease, most mechanistic studies remain small-scale and fragmented. On the clinical side, no standard treatment has been established. Chemotherapy remains the mainstay, but survival benefits are limited 5, 11, 12. Immunotherapy and targeted therapies are under exploration, yet evidence is scarce. Overall, SCCE research is still in its early phase, with significant gaps in both basic understanding and clinical management. Despite recent efforts, progress in SCCE research has been sporadic and lacks continuity. As illustrated in Figure 1, key milestones over the past decades reveal a fragmented yet gradually evolving landscape, highlighting the urgency for a unified translational perspective.

While molecular and immunological studies have shed new light on SCCE, much of the evidence remains fragmented and descriptive. Most reports are confined to limited case series or isolated mechanistic insights, making it difficult to extract clinically actionable conclusions. To date, no comprehensive review has integrated these findings to clarify their translational value. In particular, how emerging biological discoveries can inform diagnostic strategies, therapeutic selection, or future clinical trial design remains poorly articulated. This lack of synthesis continues to hinder meaningful progress in both research and patient care.

This review directly addresses that gap. From a bench-to-clinic perspective, we provide an integrated summary of recent findings on tumor biology, immune microenvironment, and molecular subtypes of SCCE. We examine current approaches, highlight unmet clinical needs, and compare SCCE with other esophageal neuroendocrine tumors to outline broader translational implications. Through this approach, we aim to offer both direction and momentum for future research into this aggressive yet underrecognized malignancy.

## 2. Molecular and Immunological Landscape of SCCE: Defining a Distinct Disease Entity

**Figure 2:**
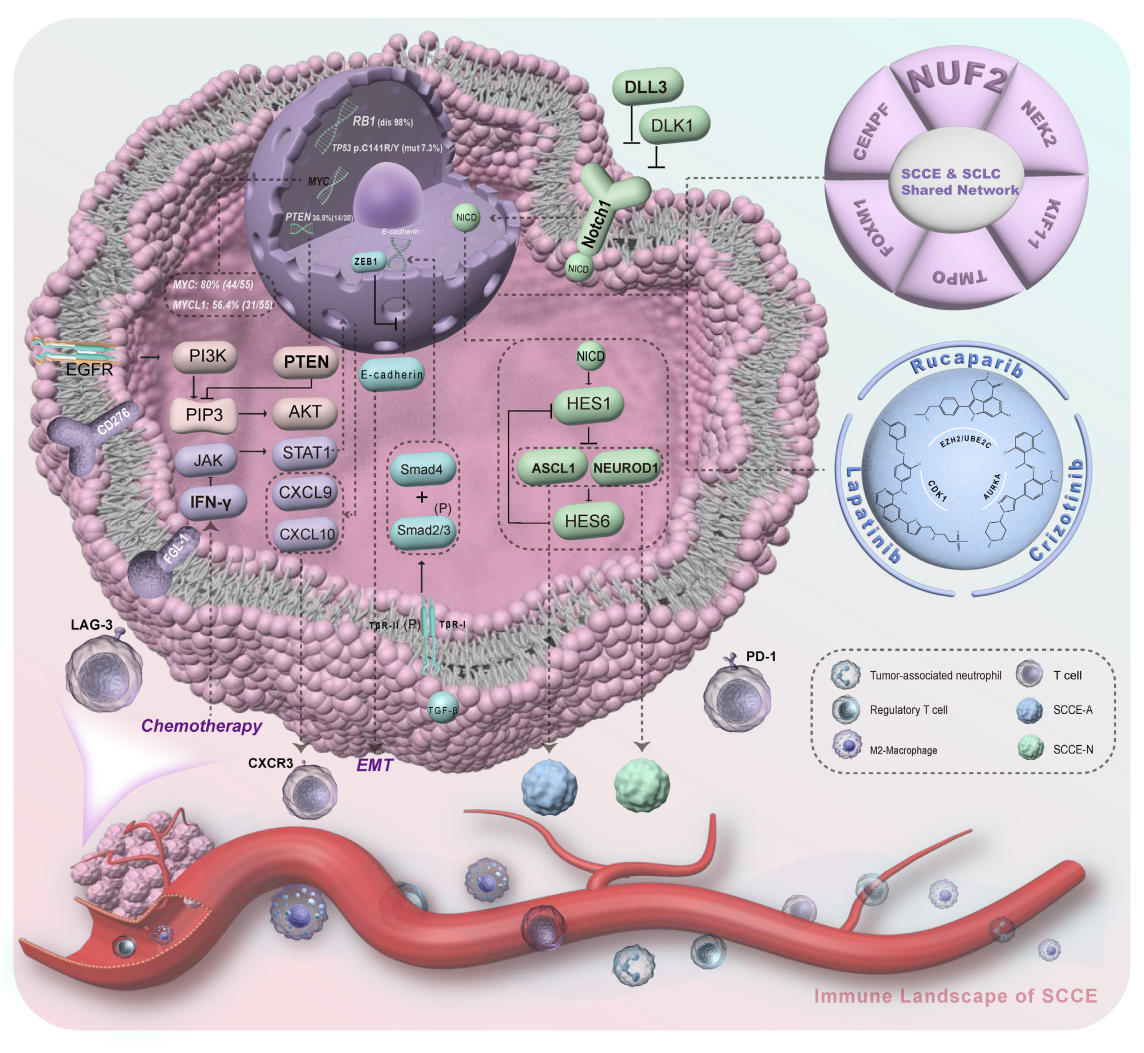
** Molecular and immune landscapes of SCCE.** This integrative schematic summarizes the key oncogenic pathways and tumor microenvironmental features of SCCE. Frequent alterations in *TP53*, *RB1*, and *PTEN*, along with copy-number gains in *MYC* and *MYCL1*, highlight the shared proliferative network between SCCE and SCLC. At the transcriptional level, *ASCL1* and *NEUROD1* define two molecular subtypes with distinct differentiation states. Loss of PTEN and activation of the PI3K/AKT and TGF-β/Smad-ZEB1 axes drive EMT and metastasis. The immune landscape is characterized by M2 macrophage infiltration, regulatory T-cell enrichment, and upregulation of inhibitory checkpoints such as PD-1, LAG-3, and CD276, indicating a strongly immunosuppressive tumor microenvironment. Additionally, chemotherapy can remodel this microenvironment by triggering the IFN-γ/STAT1-CXCL9/10 axis to recruit CXCR3+ T cells. Together, these molecular and immune circuits define SCCE as a highly proliferative, immune-cold malignancy with potential vulnerabilities in the PI3K pathway and immunoregulatory signaling. **Note:** SCCE-A, ASCL1-dominant subtype, characterized by enrichment in cell-cell adhesion and epidermal differentiation programs; SCCE-N, NEUROD1-dominant subtype, showing activation of cell cycle and protein metabolism pathways (molecular subtypes as defined in Ref 8). Abbreviations: SCCE, esophageal small cell carcinoma; SCLC, small cell lung cancer; EMT, epithelial-mesenchymal transition; NICD, Notch intracellular domain; IFN-γ, interferon gamma; TGF-β, transforming growth factor beta; PI3K, phosphoinositide 3-kinase; AKT, protein kinase B; PD-1, programmed cell death protein 1; LAG-3, lymphocyte-activation gene 3.

For a long time, the true biological identity of SCCE has been masked by its clinical presentation. Systemically, the tumor displays a neuroendocrine phenotype. At the transcriptomic level, it shares striking overlaps with SCLC across key pathways like mitosis, DNA repair, and telomere maintenance. Driven by shared core proliferative regulators such as NUF2, CENPF, and FOXM1, SCCE is frequently managed as an ectopic variant of SCLC, routinely borrowing the same chemotherapy regimens 7, 8, 13. Anatomically, the tumor follows a different pattern. SCCE typically grows beneath the mucosa and is often overlaid by synchronous squamous cell neoplasia. This spatial coexistence makes biopsy misdiagnosis a constant threat. It has also led some to classify SCCE as merely a poorly differentiated variant of esophageal squamous cell carcinoma (ESCC) 14, 15. But multi-omics sequencing is now clearing this confusion. SCCE is not a simple derivative of either malignancy. Instead, it stands as a distinct disease entity, possessing its own evolutionary trajectory and unique therapeutic vulnerabilities. Figure 2 provides a comprehensive visual map of this molecular and immunological landscape, illustrating the core genomic alterations, transcriptional networks, and microenvironmental dynamics that dictate its unique therapeutic vulnerabilities.

### 2.1 Distinct Genomic Features and Developmental Origins

Whole-exome sequencing draws a clear boundary: SCCE is genomically independent. It does share the hallmark inactivation of *TP53* and* RB1* with SCLC. Yet, a 55-sample cohort study reveals a uniquely wired landscape. The tumor harbors frequent mutations in *NOTCH1* and *PTEN*. These are closely accompanied by copy number amplifications in *MYC*, *MYCL1*, and *PIK3CA*, alongside deletions in* RB1* and *PTEN* 6.

When evaluating clonal evolution, SCCE exhibits a lower mutational burden and a simpler clonal architecture than SCLC 16. This genomic "simplicity" offers a critical clue to its origins. ESCC typically arises through a gradual, inflammation-driven progression from hyperplasia to carcinoma, steadily accumulating complex mutations. SCCE bypasses this prolonged evolutionary window. Its streamlined clonal structure points to a direct origin from multipotent stem cells in the esophageal basal layer. Here, its highly aggressive neuroendocrine differentiation trajectory is locked in at the very inception of tumor development.

### 2.2 Transcriptional Reprogramming and Invasive Drivers

Transcriptomically, SCCE completely breaks away from the esophageal squamous lineage. A recent multi-omics clustering analysis of 38 primary cases confirms that SCCE is governed by two distinct programs: one driven by ASCL1 and the other dominated by NEUROD1. This internal heterogeneity mirrors the classic molecular subtypes found in SCLC 8. Crucially, these signatures are not exclusive to the lung or esophagus. A pan-cancer study of over a thousand neuroendocrine carcinoma (NEC) samples across 31 organs reveals a striking "molecular convergence" that transcends anatomical boundaries 17. Regardless of where the tumor originates, its core biology eventually converges into fixed phenotypes defined by a few key transcription factors. This highlights a fundamental truth: ASCL1 and NEUROD1 are not specific labels for SCLC. Instead, they represent a shared evolutionary code for the entire NEC family. SCCE does not exhibit these traits because it is an "ectopic SCLC." Rather, esophageal progenitor cells independently activate this universal neuroendocrine engine during malignant transformation. Clarifying this "homologous but distinct" logic allows us to finally explore SCCE's evolutionary fate within the specific context of the esophageal microenvironment.

Biological evidence positions ASCL1 as the primary architect of early neuroendocrine phenotypes 18. A large real-world cohort of 600 pan-gastrointestinal neuroendocrine tumors from Taiwan grounds this clinically: high ASCL1 expression tightly tracks with high-grade (G3), poorly differentiated malignant states 19. The NEUROD1+ subtype charts a different course. It frequently harbors MYC amplifications 8. Beneath the surface, its transcriptional network is governed by EBF3, a medulloblastoma-associated cancer stem cell factor 17. Within SCCE-specific sequencing cohorts, this lethal combination of high stemness and MYC amplification points directly to a markedly worse clinical prognosis 8.

Yet, assessing the prognostic value of these molecular subtypes reveals an objectively complex reality. Small cohorts of poorly differentiated NECs once hinted at a localized positive correlation between high NEUROD expression and overall survival (OS)^18^. But broader evidence rewrites this narrative. In the aforementioned 600-case, pan-grade cohort—as well as in independent analyses of the downstream target DLL3—neither ASCL1, NEUROD1, nor their related surface antigens emerged as independent predictors for OS or progression-free survival (PFS) 20. This points to a crucial shift in clinical perspective. In highly heterogeneous NECs, the core translational value of molecular subtyping does not lie in merely forecasting natural survival. Instead, it serves to map specific invasion patterns and expose therapeutic vulnerabilities.

Unique transcriptional reprogramming fundamentally defines SCCE's invasiveness. NEUROD1 and EBF3 drive a pronounced high-stemness state. Such stemness firmly couples with ZEB1. As a core EMT transcription factor, ZEB1 shows striking overexpression in SCCE nuclei 21. The resulting active EMT machinery functions in profound synergy with overexpressed cell cycle engines. Core hubs like AURKA, EZH2, and CDK1 fuel this distinct progression pattern. Molecularly, these dynamics resolve a key biological paradox. SCCE harbors fewer overall driver mutations. Yet, the tumor achieves deep tissue penetrance and extremely early hematogenous dissemination. Its invasive capacity far exceeds conventional ESCC.

### 2.3 Dual-Layer Remodeling of the Immune Microenvironment

Tumor microenvironment (TME) features dictate immunotherapy responses. ESCC immune evasion typically relies on the classic PD-1/PD-L1 axis. SCCE evolves a completely different suppressive network. Bulk RNA data reveal broad immunosuppression in SCCE. M2 macrophages heavily infiltrate the microenvironment. The tumor relies primarily on upregulated alternative checkpoints like LAG-3 and CD276 22. Single-cell transcriptomics push this understanding further. Angiogenesis-driven EC1 niches and regulatory T cells (Tregs) dominate the SCCE ecosystem 9. Abnormal EC1 vascular networks form a physical barrier. Chemical suppression from M2 macrophages and alternative checkpoints interweaves with this structure. Together, these elements forge a dual-layer immune defense. Such architectural complexity clearly explains the limited clinical benefit of PD-1 monotherapy in SCCE.

### 2.4 Chemotherapy-induced Dynamic Remodeling of the Immune Microenvironment

In systemic therapy, first-line regimens like platinum-etoposide doublets go beyond traditional "pure cytotoxicity." They act as critical microenvironmental "remodelers" in highly proliferative NECs 23. Chemotherapy-induced genotoxic stress does more than kill tumor cells directly. It triggers a profound reprogramming of the immune ecology through precise molecular cascades. Specifically, DNA double-strand breaks and the accumulation of cytosolic DNA fragments activate the intracellular cGAS-STING pathway, driving the early release of type I interferons. Acting as an amplifier, this initial signal prompts immune cells within the microenvironment to secrete massive amounts of type II interferon (IFN-γ) 24-28. As the core integrator of this remodeling network, IFN-γ binds to its receptor and activates Janus kinase (JAK), leading to the phosphorylation and nuclear translocation of the key transcription factor STAT1. The full activation of the IFN-γ/STAT1 signaling axis lays the transcriptional foundation for converting the tumor microenvironment from "cold" to "hot" 29.

Among STAT1 downstream targets, the surge of CXCL9 and CXCL10 serves as the dominant force recruiting effector T cells 30. CXCL10, heavily secreted by damaged tumor and endothelial cells, establishes a robust chemotactic gradient that draws peripheral lymphocytes into the tumor bed 31. Meanwhile, IFN-γ/STAT1-dependent CXCL9 provides a localized anchoring signal, driving infiltrating CD8+ T cells to differentiate into tissue-resident memory T cells (TRM) 32. Both chemokines bind to the CXCR3 receptor on effector T cells, forming a continuous positive feedback loop: DNA damage—IFN-γ/STAT1 activation—CXCL9/10 secretion—CXCR3+ T-cell infiltration 30, 33. Yet, this microenvironmental remodeling is a double-edged sword. While driving chemokine transcription, STAT1 simultaneously acts as a key transcriptional activator for immunosuppressive molecules like PD-L1 (CD274) and IDO1 24, 34, 35. Consequently, T cells recruited by CXCL9/10 arrive at the tumor only to be rapidly exhausted by the PD-L1 "molecular brake," adaptively upregulated by the exact same pathway. This dynamic constitutes the core contradiction within the post-chemotherapy microenvironment.

### 2.5 Translating Biological Characteristics into Therapeutic Vulnerabilities

Clarifying the aforementioned molecular and microenvironmental specificities is fundamental to identifying the "therapeutic vulnerabilities" of SCCE (i.e., the dependency pathways for tumor maintenance) and informing clinical translation.

First, in the context of targeted therapies, a small cohort study of 38 Chinese patients with SCCE identified a PTEN mutation rate of 36.8%, markedly higher than that reported in other esophageal cancer histologic subtypes (e.g., ESCC) 36. This finding implies that the PI3K/AKT pathway may represent a crucial survival dependency for a subset of patients with SCCE. In parallel, molecular docking studies indicate that existing drugs—such as crizotinib, lapatinib, and rucaparib—can effectively bind and inhibit hyperactive cell cycle targets like AURKA and EZH2, highlighting their potential for drug repurposing 37.

Crucially, molecular subtypes provide precise roadmaps for next-generation targeted therapies. Among these, the ASCL1/DLL3 axis shows the greatest translational promise. In digestive small-cell NECs, ASCL1 is more than a basic transcriptional signature. It is a highly promising predictive biomarker. Specifically, DLL3 is expressed in 100% of ASCL1-positive tumors, compared to just 56.8% in the negative group. Furthermore, these two markers show high spatial concordance on the same tumor cells 20. Objective data show that high DLL3 expression does not directly prolong OS or PFS. However, this highly penetrant surface antigen enrichment provides a strong histological rationale. It supports prioritizing DLL3-targeted agents—such as the bispecific antibody tarlatamab—for ASCL1+ patients. Additionally, certain molecular subtypes exhibit primary chemoresistance. A clear example is the HNF4A-driven pan-cancer H subtype 17. This evidence further highlights the critical need for treatment triage based on transcriptomic profiling.

Second, regarding immune microenvironment regulation, single-agent PD-1 blockade cannot breach the dual-layered defense network of SCCE. Anti-angiogenic interventions address the first layer: the physical barrier of the EC1-type aberrant vascular network. By promoting tumor "vascular normalization," these therapies mechanistically facilitate the deep infiltration of effector T cells. The second layer involves alternative checkpoints like LAG-3 and CD276, on which SCCE heavily relies. Because of this dependency, single-target blockade readily triggers compensatory immune escape. Therefore, exploring combination modalities—such as anti-angiogenesis plus immunotherapy or multi-target blockade (e.g., PD-1/LAG-3)—is crucial. These approaches can synergistically reverse multiple inhibitory signals. Ultimately, mechanism-driven combination strategies are biologically far more rational than blindly extrapolating single-agent regimens from other malignancies.

Ultimately, optimizing dynamic intervention strategies requires a deep understanding of chemotherapy-induced immune remodeling. This profiling provides the theoretical foundation for defining the "optimal therapeutic window”. Single-cell evidence reveals that chemotherapy disrupts key immune pathways, such as the TIGIT-PVR and MDK-SDC4 axes 10. This disruption must be evaluated alongside the previously discussed IFN-γ/STAT1 cascade. Chemotherapy triggers a surge in CXCL9/10, driving a massive influx of T cells. This creates a "golden window" before these effector cells are completely exhausted by PD-L1. Administering immune checkpoint blockade precisely within this timeframe is critical. It removes the compensatory brake imposed by STAT1, converting a transient chemotactic advantage into durable cytotoxic efficacy. Furthermore, clinical cohorts demonstrate a clear chemotherapy-induced antigen enrichment. In pan-digestive NEC patients, prior chemotherapy exposure significantly upregulates DLL3 expression (67.2% vs. 50.5%) 20. This phenomenon provides a strong biological rationale for sequentially utilizing DLL3-targeted agents as salvage therapy following resistance to standard platinum-based regimens. Additionally, the host genetic background modulates this remodeling process. For example, patients harboring specific DNA repair gene polymorphisms (e.g., PARP1-Val762Ala) achieve extended progression-free and overall survival following chemoradiotherapy 38. Accurately capturing these dynamic shifts in both chemokine release and antigen expression will dictate the critical timing for combined immune and targeted interventions.

Altogether, SCCE is no longer just a rare neuroendocrine oddity. It's a shape-shifter—marked by molecular subtypes, shifting immune profiles, and the surprising ability to be rewired by therapy. Table 1 pulls these threads together: from driver genes to immune crosstalk, from detection to disruption. The map is still incomplete, but the direction is clear—understand its code, target its weaknesses, and exploit its plasticity. The era of decoding SCCE has begun.

## 3. Clinical Management of SCCE: Past, Present, and Prospects

**Figure 3:**
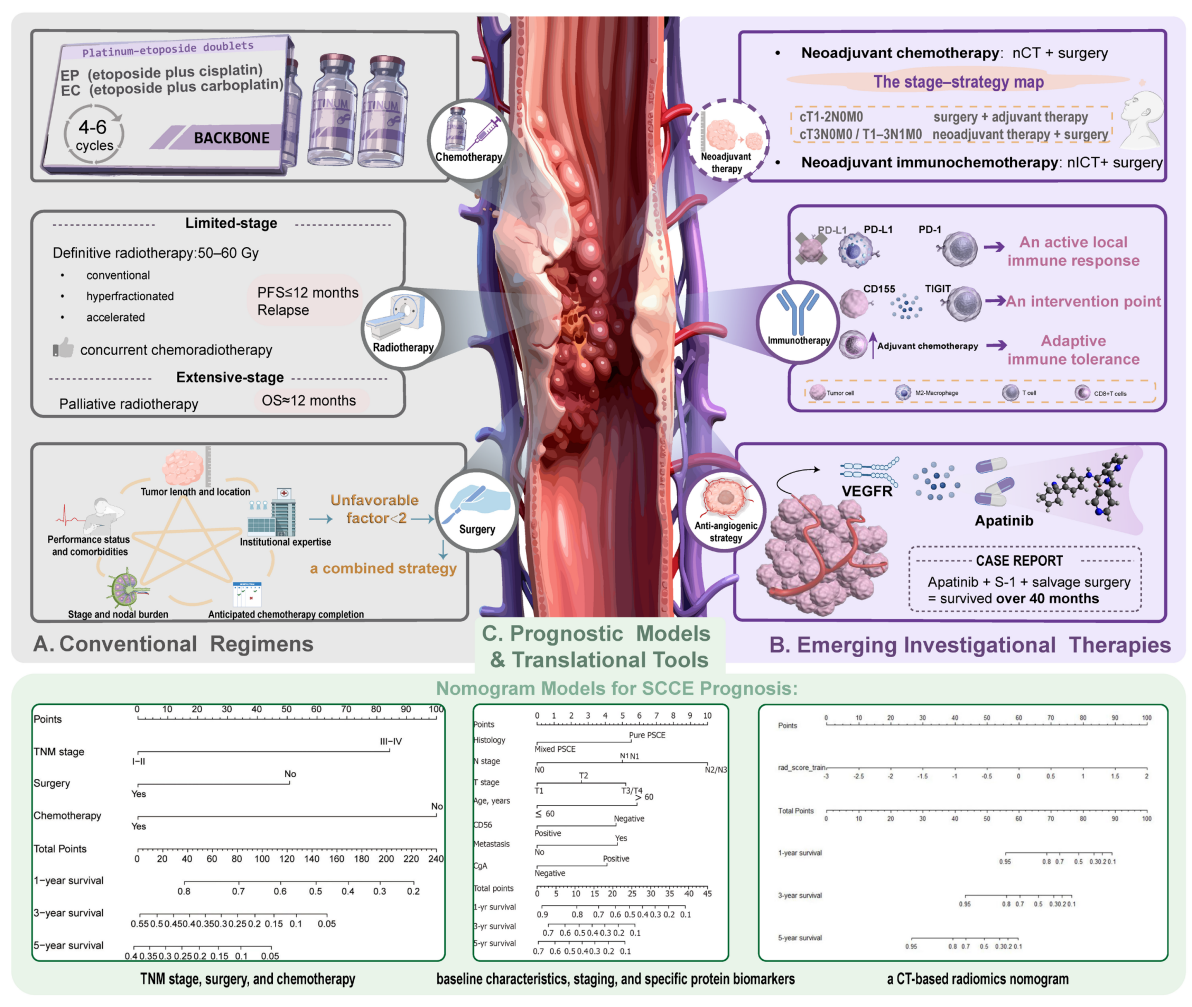
** Evolving clinical management landscape of SCCE.** This schematic illustrates the transition of SCCE treatment strategies from conventional regimens to emerging therapies and precision-oriented prognostic tools. **(A)** Conventional regimens remain anchored by platinum-etoposide doublets, specifically etoposide plus cisplatin (EP) or etoposide plus carboplatin (EC), combined with radiotherapy (50-60 Gy). Chemotherapy typically involves 4-6 cycles, achieving rapid responses but limited durability (median PFS ≤ 12 months, OS ≈ 12 months). Local treatment is tailored to stage, nodal burden, tumor location, and patient fitness. **(B)** Emerging investigational approaches emphasize biologic alignment and systemic control. Neoadjuvant chemotherapy (nCT) or immunochemotherapy (nICT) followed by surgery improves outcomes in resectable disease. Anti-angiogenic strategies (e.g., apatinib + S-1 + salvage surgery) and immune modulation through PD-1/PD-L1 and TIGIT/CD155 pathways highlight opportunities to convert “adaptive immune tolerance” into active immune response. **(C)** Prognostic and translational tools are reshaping individualized care. Composite clinical models (Tumor Node Metastasis (TNM) + surgery + chemotherapy) and CT-based radiomics nomograms collectively enable risk stratification beyond TNM staging, bridging biological understanding with clinical decision-making. [TNM stage, surgery, and chemotherapy] Reproduced with permission from Frontiers Media S.A. publisher (journal citation 105, under the CC BY 4.0 license (*https://creativecommons.org/licenses/by/4.0/*)). [baseline characteristics, staging, and specific protein biomarkers] Reproduced with permission from Baishideng Publishing Group Inc. publisher (journal citation 90, under the CC BY-NC 4.0 license (*http://creativecommons.org/Licenses/by-nc/4.0/*)). [a CT-based radiomics nomogram] Reproduced with permission from Frontiers Media S.A. publisher (journal citation 91, under the CC BY 4.0 license (*https://creativecommons.org/licenses/by/4.0/*)). Abbreviations: SCCE, esophageal small cell carcinoma; EP, etoposide and cisplatin; EC, etoposide and carboplatin; PFS, progression-free survival; OS, overall survival; nCT, neoadjuvant chemotherapy; nICT, neoadjuvant immunochemotherapy; PD-1, programmed cell death protein 1; PD-L1, programmed death-ligand 1; TIGIT, T cell immunoreceptor with Ig and ITIM domains; VEGFR, vascular endothelial growth factor receptor; TNM, tumor-node-metastasis; CT, computed tomography; CD56, cluster of differentiation 56; CgA, chromogranin A.

Despite growing awareness of its unique biological identity, the clinical management of SCCE remains largely tethered to borrowed standards. Most treatment regimens still mirror those for small-cell lung cancer, with little adaptation to the esophageal context. Yet mounting clinical observations—ranging from variable response rates to differing relapse patterns—have begun to challenge this one-size-fits-all approach. To illustrate this evolution more clearly, Figure 3 summarizes the shifting landscape of SCCE management, tracing the trajectory from conventional regimens to emerging therapies and future directions. This section further unpacks these changes in detail.

### 3.1 Conventional Regimens

#### 3.1.1 SCLC Regimens in SCCE: Borrowed Logic, Questionable Fit

In the absence of disease-specific guidelines, it was almost inevitable that treatment for SCCE would follow the well-worn path of SCLC. Both share a neuroendocrine phenotype, rapid proliferation, and early dissemination—features that made the SCLC template an attractive ready-made solution. In practice, the backbone has been platinum-etoposide doublets—most commonly EP or EC—delivered over 4 to 6 cycles 11, 39, 40. For patients with limited-stage disease, these regimens are typically paired with definitive radiotherapy, often in the range of 50-60 Gy, echoing SCLC protocols 41-43. The rationale was straightforward: achieve rapid cytoreduction with chemotherapy and then consolidate local control with radiation.

The early results seemed to justify the borrowing. Retrospective analyses in limited-stage SCCE frequently documented objective response rates above 70%, with rapid relief of dysphagia and improvement in swallowing function 3. However, the critical differences in treatment durability and prognosis soon became clear. Median PFS rarely extended beyond 12 months, and relapses—both locoregional and distant—were common 43, 44. In extensive-stage disease, systemic therapy could still shrink tumors, but the median OS stubbornly hovered around one year, a survival profile closely resembling that of SCLC 3, 45, 46. Yet, beneath these shared dismal outcomes, the clinical trajectories diverge. That reality now shapes how we use radiation. In fit, limited-stage patients, it anchors local control, typically alongside chemotherapy. When disease is advanced—or patients are frail—the same tool turns palliative: easing swallowing, controlling bleeding, preventing obstruction 47-50. And in sharp contrast to SCLC's prognostic management, prophylactic cranial irradiation (PCI) is seldom used, given fewer brain metastases and uncertain benefit in SCCE 43, 51, 52.

What emerges from these decades of practice is a mixed picture: the SCLC blueprint delivers fast, visible responses, but its durability is limited. Even in the best responders, systemic failure casts a long shadow, exposing the shortcomings of a simple therapeutic transplant from lung to esophagus. This has prompted a quiet but persistent question within the field: if SCCE is not SCLC, why are we still treating it as if it were?

#### 3.1.2 Radiotherapy, Chemoradiotherapy, or Surgery: A Shifting Balance

In the history of SCCE treatment, the SCLC model offered a “quick-response” solution—tumors shrank fast, symptoms eased quickly, but relapses came just as swiftly. As these limitations became clear, clinical attention shifted: which local approach holds greater value? Should we rely on the synergy of radiotherapy and chemoradiotherapy, or choose surgery decisively in selected patients? The answer is not fixed, but continually rebalanced according to disease stage and patient fitness.

After moving beyond the SCLC model, radiotherapy—often with concurrent chemotherapy—has become a core local treatment for SCCE. A nationwide cohort added important context: in-field recurrence was rare with 45-50 Gy, but survival did not rise proportionally with dose. The real difference came from chemotherapy intensity—patients receiving ≥4 cycles had the most consistent benefit. Most relapses occurred within the first year and were predominantly distant 43. Registry-based data echoed this finding, showing radiotherapy was linked to better survival, especially in limited-stage cases 53. A China-U.S. comparative analysis added nuance: stage distribution and treatment patterns varied markedly—Chinese patients more often had limited disease and received chemoradiotherapy, whereas U.S. patients presented at more advanced stages and were more likely to receive radiotherapy alone. After propensity-score matching, overall survival differences narrowed, suggesting outcomes were driven less by geography than by stage composition and treatment intensity 54. Retrospective studies and meta-analyses consistently reinforce the survival benefit of radiotherapy hinges on adequate systemic therapy and appropriate patient selection 5, 55-58.

Case reports and small series illustrate these patterns in real-world patients. EP/EC or cisplatin plus irinotecan with 45-60 Gy has produced complete imaging and endoscopic responses: some elderly or frail patients achieved long-term, recurrence-free survival 59; others with locally advanced disease maintained durable remission after S-1/cisplatin with 60 Gy 60. In one definitive concurrent chemoradiotherapy (dCRT) series for resectable SCCE, the complete response rate was 100%, with a median survival of 32 months and no major late toxicity, though acute hematologic toxicity was common 61. Still, complete response on scans does not mean systemic cure. Some patients developed brain or liver metastases months after local remission, highlighting that systemic escape is the major hurdle 44, 49.

For extensive-stage disease, chemotherapy remains central. A retrospective series of irinotecan plus cisplatin showed a 50% response rate but a median OS of only 12.6 months 62. Here, local radiotherapy serves mainly as selective consolidation within systemic treatment, rather than as a standalone solution.

The overall message is clear: for limited-stage, non-surgical candidates, high-quality concurrent chemoradiotherapy should be the default. Reliable in-field control is achievable with 45-50 Gy, but survival gains hinge on delivering enough chemotherapy—four or more cycles provide more stable outcomes. Effort should focus on delivering full systemic treatment and identifying patients most likely to benefit from local intensification, such as those with good performance status and small tumor burden. Whether and when to introduce surgery then depends on how much systemic treatment benefit this platform can secure 12, 47, 61, 63. The next step is to examine surgical strategies and their integration with chemoradiotherapy.

For years, SCCE was regarded as a “systemic small cell carcinoma,” and the role of surgery was undervalued. Yet several surgical series have challenged this view: in resectable or limited-stage patients, median OS typically ranged from 18 to 27 months 64-66, with occasional long-term survivors free of recurrence 67, 68. This benefit follows identifiable patterns: patients with negative lymph nodes (pN0), tumors in the lower thoracic esophagus or gastroesophageal junction (EGJ), and tumors <5 cm define a relatively clear “sweet spot” for surgery 65, 69.

Surgery alone is rarely optimal. Multiple studies consistently showed better outcomes with surgery plus chemotherapy: OS was 21.0 months with surgery plus chemotherapy versus 14.1 months with surgery alone; in 73 limited-stage patients, median survival was 27.0 months with postoperative chemotherapy and 13.0 months without 65, 69. Another series also identified radical resection and chemotherapy as independent prognostic factors 70. Further stratification suggests that postoperative chemotherapy significantly improves survival in limited-stage II or "surgery-responsive disease" (SRD), whereas the incremental value of postoperative chemoradiotherapy in completely resected stage II cases remains questionable 71-75. In esophageal neuroendocrine carcinoma, a similar stratification showed that adjuvant therapy significantly benefited surgery-responsive limited disease (SRLD) patients but not the surgery-non-response limited disease (SNRLD) group 76. Both frameworks emphasize matching surgery to stage and risk profile, rather than treating it as a universal solution. Furthermore, retrospective analyses show that greater tumor length and depth of invasion are strongly associated with higher risk of lymph node metastasis 77. Precision in applying surgery hinges largely on nodal status. In NCDB and SEER data, patients with limited-stage, node-negative disease (T1-4aN0M0) achieved the longest survival with esophagectomy (median OS 44.9 months) 78, 79. In contrast, for node-positive localized disease, definitive chemoradiotherapy appeared more favorable than surgery plus chemotherapy 80. These findings support tailoring local treatment to nodal burden rather than applying a single standard across all limited-stage patients.

Taken together, in local treatment for SCCE, the priority is whether the approach aligns with tumor biology and the achievable intensity of systemic therapy. Because postoperative pathology frequently reveals occult nodal metastases even in clinically "limited" disease, surgery must be integrated into a “systemic control first” paradigm rather than positioned as a standalone solution. High-quality concurrent chemoradiotherapy should be the default if nodal burden is suspected, tolerance is limited, or anatomy makes complete resection uncertain. Radiotherapy (45-50 Gy) secures in-field control, but simply increasing the radiation dose cannot replace systemic control. Conversely, for carefully selected limited-stage SCCE (pN0, lower/EGJ location, small tumor volume), radical esophagectomy combined with perioperative systemic therapy yields net benefit. Final decision-making can be distilled into five levers: stage and nodal burden, tumor length and location, performance status, anticipated chemotherapy completion, and institutional expertise. Building upon these conventional clinical levers, Table 2 presents a comprehensive evidence-grading and stratification framework for SCCE management. This matrix not only consolidates the established standard-of-care based on traditional staging, but also previews emerging biomarker-driven strategies (e.g., PD-L1 and DLL3), which will be systematically detailed in the subsequent section.

### 3.2 Emerging Investigational Therapies

Despite longstanding therapeutic challenges in SCCE, the tide may finally be turning. While survival remains poor and standardized guidance is still lacking, recent advances are beginning to illuminate new paths. Instead of isolated findings, a coherent body of evidence is forming—starting with accessible biomarkers like inflammatory and nutritional indices, progressing to insights into the immune microenvironment and molecular heterogeneity, and culminating in rational treatment selection and predictive modeling. These emerging layers do more than deepen complexity—they chart a course toward truly individualized care. Table 3 summarizes several promising multidimensional biological features across inflammatory, microenvironmental, molecular, and endocrine landscapes. This compilation illustrates the bench-to-bedside transition of these markers. Furthermore, it defines their specific utility in guiding the precision management of SCCE. In the sections below, we outline how each of these elements is converging to reshape the clinical and biological understanding of SCCE.

#### 3.2.1 Tumor Immune Microenvironment: Signals That Matter

Traditional paradigms often dismiss SCCE as a simple immune-"cold" tumor. However, this label obscures the highly complex dynamics operating within its microenvironment. Recent pathological evidence clarifies this complexity. The microenvironment does more than merely harbor essential prognostic and predictive signals. It fundamentally provides the mechanistic rationale for the severe limitations of single-agent immune checkpoint blockade (ICB).

The spatial heterogeneity of the PD-1/PD-L1 axis presents a critical challenge. A study analyzing 147 samples from the Chinese National Cancer Center revealed that PD-L1 expression on SCCE neoplastic cells is nearly nonexistent. Instead, PD-L1 is predominantly expressed on tumor-infiltrating immune cells, specifically identified as CD68⁺ macrophages 81. Even within a broader cohort of poorly differentiated digestive neuroendocrine carcinomas, the PD-L1 positivity rate on tumor cells remains a mere 14%. This widespread immune marker "silence" among tumor clones essentially reflects an inherent defect in antigen presentation. Crucially, it exposes the primary mechanism behind the failure of single-agent therapies: impaired immune recognition. Consequently, even if PD-1 blockade successfully relieves T-cell suppression, the effector cells remain functionally "blind." Lacking clear recognition targets on the tumor parenchyma, these cells ultimately fail to execute precise cytotoxicity 82.

This upstream recognition deficit directly constrains the pre-existing immune potential within the microenvironment. Multiple studies confirm the clinical value of CD8⁺ tumor-infiltrating lymphocytes (TILs) in SCCE tissues. A higher density of these cells predicts both extended survival and substantial benefit from adjuvant chemotherapy 81, 83. Notably, the TCF1⁺CD8⁺ subset, characterized by stem-like self-renewal properties, emerges as the primary driver of this survival improvement. These findings indicate that a salvageable, latent anti-tumor response genuinely exists within the local microenvironment. Despite this potential, these promising T cells fail to eradicate the tumor.

Another dimension of pathological data reveals the root of this limitation. The infiltration density of CD8⁺ T cells exhibits a highly significant positive correlation with microenvironmental PD-L1 expression (P < 0.0001). This dynamic exemplifies classic "adaptive immune resistance." As effector T cells attempt to infiltrate and execute cytotoxic functions, the microenvironment mounts a rapid defense. It mobilizes surrounding macrophages to upregulate PD-L1, effectively erecting a reactive, chemical inhibitory barrier around the tumor periphery 84.

Beyond these specific adaptive responses, SCCE extensively exploits multiple co-inhibitory pathways to sustain immune evasion. Consider the increasingly recognized TIGIT/CD155 axis. Both CD155 and its receptor TIGIT exhibit marked upregulation in SCCE, a profile tightly correlated with heavier tumor burden, advanced clinical stages, and inferior survival outcomes. Against this redundant inhibitory network, single-agent PD-1 blockade proves profoundly inadequate. Under the selective pressure of single-target inhibition, the microenvironment readily orchestrates a compensatory defense by upregulating alternative checkpoints like TIGIT. This rapid adaptation relentlessly re-suppresses effector T cells just as they reach the verge of reactivation 85.

The resistance of SCCE to PD-1 monotherapy emerges from the intricate synergy of these two mechanisms. Upstream, antigen silencing within tumor clones dictates severe "impaired recognition". Downstream, the microenvironment enforces a formidable barricade, driven by adaptive resistance and multi-pathway compensatory bypasses. Deciphering this fundamental logic illuminates the path to therapeutic breakthroughs. Single-target blockade remains fundamentally inadequate. Successfully dismantling this dual immunological dilemma demands multi-dimensional, combinatorial interventions.

#### 3.2.2 Overcoming Immune Evasion: Theoretical Rationale and Clinical Exploration of Combination Strategies

Driven by the dual limitations of upstream "impaired recognition" and downstream "compensatory bypasses" that restrict single-agent PD-1/PD-L1 blockade in SCCE, clinical intervention strategies must inevitably evolve toward multi-dimensional combination therapies. These approaches aim to remodel the tumor microenvironment across multiple dimensions to overcome monotherapy resistance.

A primary focus is the combination of chemotherapy and immunotherapy. Large-cohort studies have definitively established the foundational role of neoadjuvant chemotherapy (nCT) in limited-stage SCCE. Analyses from Chinese multicenter cohorts and the SEER database yield consistent results. For patients with locally advanced disease (e.g., cT3N0M0 or node-positive), nCT followed by surgery significantly improves both overall survival (OS) and disease-free survival (DFS) 86-89. Integrating immunotherapy into this baseline regimen rests on a robust mechanistic rationale. Beyond exerting direct cytotoxic effects, chemotherapeutic agents induce immunogenic cell death (ICD). This specific process amplifies the release of tumor neoantigens, effectively reversing the previously described "impaired recognition." Recent clinical explorations provide preliminary support for this strategy. Patients with locally advanced SCCE receiving neoadjuvant immunochemotherapy (nICT) achieved durable clinical remissions. Histological analyses further confirm that this therapeutic efficacy closely correlates with high baseline CD8⁺ T-cell infiltration and PD-L1 expression 90. These findings prove that chemotherapy successfully exposes tumor antigens, providing explicit targets for subsequent checkpoint blockade.

Another critical approach involves combining anti-angiogenic agents with immunotherapy. The rapid proliferation of SCCE relies heavily on an aberrant microvascular network. This structural abnormality severely restricts conventional drug delivery. It also constructs a formidable physical barrier against the deep infiltration of effector T cells. Clinically, targeting angiogenesis shows proven potential in refractory cases. For instance, apatinib salvage therapy has yielded prolonged survival 91. Introducing anti-angiogenic drugs serves as a vital component of combinatorial interventions. These agents effectively sever the tumor's metabolic supply lines. More importantly, they drive the "normalization" of aberrant microvessels, alleviating local hypoxia and clearing physical conduits for T-cell infiltration. This profound remodeling of the microenvironment establishes optimal conditions for immunotherapeutic engagement. Recent clinical explorations substantiate this mechanistic rationale. One report details an advanced SCCE case that developed secondary resistance to single-agent anti-angiogenic therapy (e.g., anlotinib). The synchronous addition of PD-1 blockade (e.g., toripalimab) successfully reversed this resistance, inducing a complete response (CR). Notably, this deep clinical response coincided with specific molecular signatures, including microsatellite instability (MSI) and a high tumor mutational burden (TMB-H) 92. While currently derived from case-level evidence, these findings offer a compelling proof of concept. The "vascular remodeling plus immune activation" paradigm clearly demonstrates the potential to overcome monotherapy resistance. Moving forward, leveraging next-generation sequencing (NGS) tools will be essential to precisely define the populations most likely to benefit from these joint strategies.

A further strategy involves multiplex immune checkpoint blockade. Single-agent interventions frequently provoke "adaptive compensation" within the tumor microenvironment. Countering this phenomenon necessitates synchronously targeting multiple co-inhibitory pathways, offering a rational approach to overcome secondary resistance. As previously established, SCCE exhibits widespread overexpression of alternative bypasses, particularly the TIGIT/CD155 axis. This biological reality dictates that isolated PD-1 blockade readily triggers the compensatory upregulation of other checkpoints. Addressing this challenge requires dual intervention regimens. Strategies that simultaneously block PD-1 alongside TIGIT or LAG-3 aim to systematically dismantle the redundant inhibitory network within the microenvironment. These comprehensive approaches fundamentally mitigate the incidence of secondary resistance.

Together, combination therapy is not an arbitrary accumulation of clinical drugs. Instead, it represents a highly targeted intervention designed to dismantle the specific immune defense mechanisms of SCCE. Several mechanism-driven strategies now constitute the core theoretical framework for future prospective clinical trials. These encompass chemotherapy-mediated antigen release, anti-angiogenic-driven microenvironmental remodeling, and the suppression of compensatory pathways through dual checkpoint blockade.

#### 3.2.3 Molecular and Endocrine Signatures Shaping Prognosis and Therapy in SCCE

The rapid evolution of mechanism-driven combination therapies necessitates precise clinical stratification tools. Beyond traditional TNM staging, biomarkers have transcended their role as mere laboratory readouts. They now fundamentally reshape our prognostic frameworks and actively dictate therapeutic pathways. Ultimately, the clinical trajectory of SCCE defies the simplistic paradigm that greater aggressiveness equates to a poorer prognosis.

Consider how DNA repair capacity shapes treatment outcomes. A specific polymorphism in the *PARP1* gene (Val762Ala) has been linked to longer survival and better response to radiochemotherapy—but only in SCCE 38. Interestingly, patients carrying multiple “favorable” genotypes fared even better. Clinically, this implies that DNA repair profiles may soon help guide who gets what—and who might actually benefit. Then comes Ki-67, long feared as a marker of tumor aggressiveness. But SCCE rewrites that script. Here, a high Ki-67 index doesn't mean doom—in fact, it's been associated with better outcomes and stronger benefit from adjuvant therapy 93. What looks dangerous might actually mark tumors that are more responsive to treatment. Neuroendocrine markers like CgA aren't just diagnostic props—they're prognostic tools. Patients with CgA-positive tumors live longer, according to both broad cohorts and studies focused on early-stage disease 94, 95. We've been underestimating these “old” markers. It's time to bring them back into the conversation. Beyond CgA, other neuroendocrine markers long applied in small cell lung cancer are gaining recognition in SCCE 96. Serum neuron-specific enolase (NSE) and pro-gastrin-releasing peptide (ProGRP), especially in combination, show strong diagnostic performance against ESCC and esophageal adenocarcinoma (EAC). More importantly, their dynamic changes during treatment and follow-up mirror disease control or progression, and high levels predict shorter PFS and survival 97. Together with CgA, these markers underscore the prognostic and monitoring value of neuroendocrine signatures in SCCE. And then there's Lgr5. This stemness-related protein isn't just a bystander. When Lgr5 is high, trouble follows—with more lymph node spread, later stage at diagnosis, and weaker chemo responses 98.

#### 3.2.4 Clinical Prognostic Significance of Inflammatory and Nutritional Markers

Beyond microscopic molecular signatures, inflammatory and nutritional indices reflecting the systemic host status are equally critical dimensions for prognostication. In SCCE, several inflammation-related markers have stood out as simple yet powerful predictors of survival. One of the most consistent signals comes from the platelet-to-lymphocyte ratio (PLR). High PLR levels have been repeatedly linked to worse overall survival, even after adjusting for other clinical factors 99. Notably, its prognostic performance appears to outperform the more commonly studied neutrophil-to-lymphocyte ratio (NLR), suggesting that platelet-driven immune responses may play a distinct and critical role in disease progression 100. C-reactive protein (CRP), a classic marker of systemic inflammation, also shows strong clinical relevance. Elevated CRP levels are associated with more advanced T and N stages and are tightly correlated with shorter overall survival, making it a potential surrogate for tumor burden and immune dysregulation 101. More recently, the albumin-to-fibrinogen ratio (AFR) has emerged as another independent prognostic factor. Because it captures both systemic inflammation and nutritional status, low AFR levels identify patients at significantly higher risk of poor survival 102.

Beyond circulating markers, tumor-infiltrating immune cells reveal another layer of insight. In SCCE tissue, higher eosinophil infiltration has been linked to better survival outcomes, hinting at a beneficial role for certain immune cells within the tumor microenvironment 103. This contrast—where systemic inflammation signals worse prognosis while local immune activation may be protective—underscores the complexity of immune dynamics in SCCE.

#### 3.2.5 The Rise of Prognostic Models and Risk Stratification Tools

Neither microscopic molecular sequencing nor macroscopic systemic inflammation assessments alone can fully capture the profound heterogeneity of SCCE. Constrained by a purely anatomical focus, the traditional TNM staging system frequently fails to explain the starkly divergent survival outcomes among patients within the same stage, offering little guidance for personalized therapy. Consequently, the urgent demand for precision risk stratification has catalyzed a paradigm shift in prognostic evaluation. The field is now advancing beyond single-dimensional metrics, embracing integrated predictive models that deeply fuse multi-modal clinical, pathological, hematological, and imaging data.

A key early advance was the establishment of a risk stratification system based purely on clinical parameters. One such model categorized patients into four subtypes—LLD (low-risk localized disease), HLD (high-risk localized disease), LMD (low-risk metastatic disease), and HMD (high-risk metastatic disease)—with strikingly divergent 3-year OS rates ranging from 52.5% to just 5.7%, significantly outperforming TNM staging 104. Yet these early attempts, while encouraging, were constrained by small cohorts and single-institution data. That gap was recently addressed by a multicenter study of more than 490 patients, which refined the concept into the so-called TSC model. By incorporating TNM stage, surgery, and chemotherapy, this model achieved sharper risk discrimination than both TNM and Veterans Administration Lung Study Group (VASLG), with robust separation of high- and low-risk groups 105. Crucially, unlike earlier exploratory tools, it underwent both internal and external validation, making it the first truly reliable prognostic system tailored for SCCE.

Yet clinical data alone can only go so far. Attention has since turned to dynamic indicators of host response—namely, nutritional and inflammatory markers. A nomogram incorporating hemoglobin, NLR, and platelet count demonstrated a C-index of 0.728, clearly superior to TNM (0.614), and showed marked advantages in decision curve and net reclassification analyses 106. The strength of this model lies in its accessibility—these lab values are readily available, making it especially useful in real-world settings.

Building on this, some efforts have focused on integrating clinical parameters, pathological features, and molecular markers into a unified model. One retrospective study in Chinese SCCE patients developed and validated a practical nomogram that incorporated baseline characteristics, staging, and specific protein biomarkers 90. The model achieved C-index values of 0.659 and 0.700 in the training and validation cohorts, respectively, both outperforming the 7th edition TNM staging system. It also demonstrated good calibration and delivered greater net benefit in decision curve analysis, offering a more refined tool for individualized risk stratification in clinical practice.

Radiomics is increasingly proving its value in SCCE prognosis, especially for noninvasive prediction of survival outcomes. One retrospective study constructed a CT-based radiomics nomogram that predicted OS with high accuracy. The model outperformed traditional clinical models, achieving C-index values of 0.844 and 0.805 in the training and testing cohorts, respectively 91. Another multicenter study explored contrast-enhanced CT features and identified degree of enhancement, N stage, and adjuvant chemotherapy as independent predictors of both OS and PFS. Interestingly, the enhancement pattern on imaging also correlated with treatment responsiveness, particularly to adjuvant chemotherapy 107. Together, these findings highlight radiomics as a promising tool not only for baseline risk stratification, but also for identifying treatment-sensitive subgroups in SCCE.

Taken together, SCCE prognostication is undergoing a paradigm shift—from broad-stroke staging toward precision risk assessment. Whether through clinical parameters, host biomarkers, or radiomics signatures, the trajectory is clear: predictive models are moving beyond survival estimates to inform and individualize treatment itself. To systematically synthesize the treatment paradigms and clinical challenges discussed throughout this section, Table 4 outlines the specific therapeutic advantages, current bottlenecks, and future directions for reversing SCCE resistance across different modalities.

## 4. Blurred Pathological Boundaries in SCCE: Challenges and Clinical Implications

**Figure 4:**
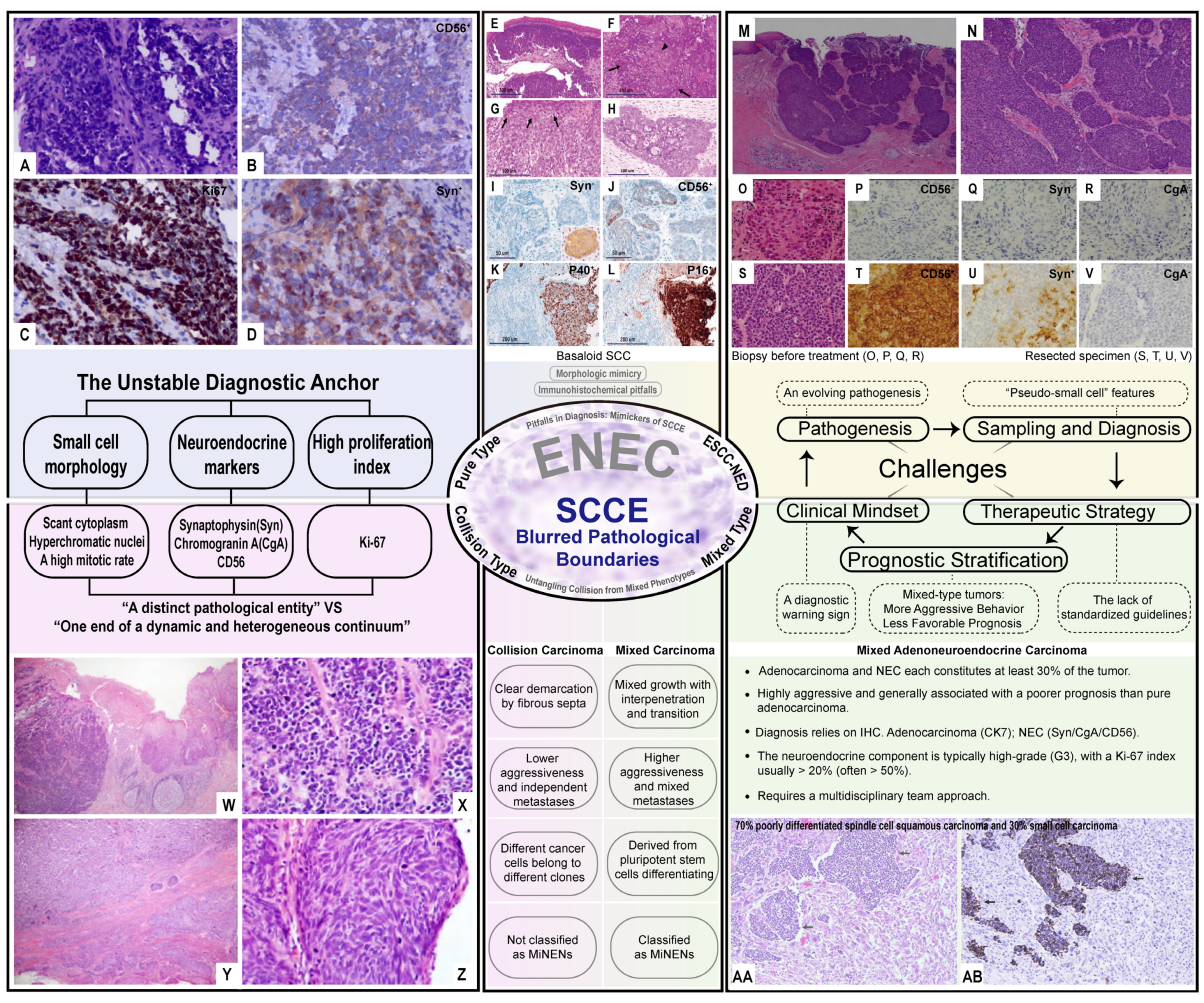
** Blurred pathological boundaries of SCCE and their clinical implications.** This schematic illustrates the morphologic and biological continuum of SCCE across the neuroendocrine spectrum. On the left, pure small cell carcinoma represents the classical form, defined by small hyperchromatic cells, scant cytoplasm, and diffuse expression of neuroendocrine markers (CgA, Syn, CD56). Toward the center, tumors often incorporate squamous or glandular elements, forming mixed-type or collision-type lesions with distinct growth patterns and variable marker overlap. To the right, basaloid squamous cell carcinoma and adenocarcinoma with neuroendocrine differentiation (ANEC) exemplify partial neuroendocrine transformation within non-neuroendocrine lineages. [A, B, C, D] Reproduced with permission from Cureus, Inc. publisher (journal citation 150, under the Creative Commons Attribution (CC-BY) license). [E, F, G, H, I, J, K, L] Reproduced with permission from Histology and Histopathology publisher (journal citation 121, under the Creative Commons CC-BY International License). [M, N, O, P, Q, R, S, T, U, V] Reproduced with permission from International Institute of Anticancer Research (journal citation 151). [W, X, Y, Z] Reproduced with permission from e-Century Publishing Corporation publisher (journal citation 112, under the Creative Commons Attribution Noncommercial License). [AA, AB] Reproduced with permission from Springer Nature publisher (journal citation 152, under the Creative Commons Attribution 4.0 International License (*http://creativecommons.org/licenses/by/4.0/*)). [Central schematic (illustrating blurred pathological boundaries)] Reproduced with permission from American Federation for Medical Research and Sage publisher (journal citation 153, under the Creative Commons Attribution-NonCommercial 4.0 License (*https://creativecommons.org/licenses/by-nc/4.0/*)). Abbreviations: SCCE, esophageal small cell carcinoma; CgA, chromogranin A; Syn, synaptophysin; CD56, cluster of differentiation 56; ANEC, adenocarcinoma with neuroendocrine differentiation; ENEC, esophageal neuroendocrine carcinoma; ESCC-NED, esophageal squamous cell carcinoma with neuroendocrine differentiation; SCC, squamous cell carcinoma; CK7, cytokeratin 7; G3, grade 3; IHC, immunohistochemistry; MiNENs, mixed neuroendocrine-non-neuroendocrine neoplasms; NEC, neuroendocrine carcinoma.

Although SCCE is usually defined by its small cell morphology, neuroendocrine markers, and high proliferation index, reality is far less clear-cut. In practice, many tumors defy strict classification, spanning a spectrum from pure small cell forms to mixed or collision types, squamous transformations, and adenocarcinomas with neuroendocrine differentiation. These blurred pathological boundaries do not merely complicate diagnosis—they directly shape therapeutic choices and survival outcomes. Figure 4 provides an overview of this spectrum and underscores the clinical challenges posed by such ambiguity, serving as a visual guide for the discussion that follows. Understanding how these ambiguities translate into clinical practice is essential for developing rational and individualized treatment strategies.

### 4.1 Esophageal Neuroendocrine Carcinomas (ENECs): Diagnostic Anchor or a Shape-Shifting Subtype in Disguise?

Among all esophageal malignancies, SCCE is undoubtedly one of the most aggressive. It typically presents insidiously, progresses rapidly, and is often diagnosed at an advanced stage. Although rare, SCCE accounts for the vast majority of ENECs. As a broader pathological category, ENECs also include large cell neuroendocrine carcinoma (LCNEC), albeit much less frequently 108. Both subtypes are classified as high-grade malignancies 108, 109. Morphologically, SCCE is characterized by scant cytoplasm, hyperchromatic nuclei, and a high mitotic rate, while LCNEC often displays prominent nucleoli and gland-like or trabecular structures 110-113. These features seem to offer a “diagnostic anchor” for ENECs. Yet in clinical reality, this anchor often feels unstable. Even tumors with seemingly “pure” morphology may harbor squamous or glandular components, or evolve into mixed or transformed phenotypes. Whether ENEC constitutes a distinct pathological entity—or simply represents one end of a dynamic and heterogeneous continuum—remains open to question.

Clinically, ENEC is known for its aggressive behavior, rapid progression, early metastasis, and dismal prognosis. However, this is not always the case. Some tumors are incidentally discovered during endoscopic examinations and can even be completely resected via endoscopic submucosal dissection 114. There are also patients who achieve long-term disease-free survival following multidisciplinary treatment 115. This clinical heterogeneity not only adds to diagnostic confusion but also challenges the notion of ENEC as a uniform disease entity.

In terms of treatment, approaches vary widely across institutions. As mentioned earlier, most centers adopt SCLC regimens, primarily platinum-based chemotherapy. Yet in practice, treatment strategies are more diverse. Some patients have experienced durable responses to immune checkpoint inhibitors combined with anti-angiogenic agents, such as camrelizumab plus apatinib 116, though large-scale prospective data are still lacking. Other studies emphasize the potential for curative outcomes in selected patients who undergo resection followed by tailored adjuvant management 117, 118. These divergent experiences reflect a deeper uncertainty about what ENEC actually represents.

At the molecular level, there is still a lack of definitive evidence to support ENEC as a biologically stable subtype. A recent study on microRNAs identified a specific miRNA signature (e.g., miR-1246, miR-1260a, miR-1260b) that was significantly associated with postoperative relapse and overall survival 117. However, these findings have yet to enter clinical application and have not meaningfully advanced molecular subtyping of ENEC. Although a consensus classification for ENEC remains to be established, the well-characterized paradigms of other neuroendocrine malignancies offer a valuable roadmap. Table 5 juxtaposes the emerging immune and molecular landscapes of SCCE against the established subtypes of gastric and lung neuroendocrine tumors, highlighting shared mechanisms and distinct therapeutic vulnerabilities.

### 4.2 Beyond ENEC: Neuroendocrine Differentiation in Squamous, Glandular, and Mixed-Type Esophageal Cancers

Although ENEC is categorized as an independent entity in the World Health Organization (WHO) classification, its identity in clinical practice is far more nuanced. Representing less than 2% of all esophageal cancers, ENEC rarely arises de novo; instead, mounting evidence shows it often evolves from squamous cell carcinoma or adenocarcinoma 14, 15, 119. This suggests ENEC may not be a distinct tumor type but rather the neuroendocrine endpoint of tumor progression. Studies frequently reveal coexisting or even dominant non-neuroendocrine components, which, if overlooked, can mislead pathology and steer treatment toward inappropriate SCLC-based regimens, bypassing options like surgery or targeted therapy 109, 120. Rather than a fixed category, ENEC should be viewed as a diagnostic warning sign—prompting deeper reflection on its origins and the need for individualized treatment strategies.

Not all neuroendocrine-like features within esophageal tumors are what they seem. Recent evidence has revealed that conventional squamous cell carcinoma (SCC) can exhibit striking neuroendocrine-like differentiation—without being a true neuroendocrine carcinoma. A prime example is basaloid SCC, which may express markers like CD56 or p16 in a patchy, misleading fashion 121. Such expression profiles can mimic small cell carcinoma, especially in small biopsy samples where architectural context is limited. Misdiagnosis becomes a real threat when pathologists rely solely on morphology, particularly in cases with “pseudo-small cell” features but preserved p40 positivity—a hallmark of squamous lineage. Immunohistochemistry, therefore, is not just supplementary, but essential in avoiding diagnostic traps. Building on these blurred histologic lines, adenocarcinoma too is no exception. Neuroendocrine differentiation can emerge within glandular tumors, often going unnoticed under routine evaluation 122. Among these, ANECs represent a particularly elusive subtype-rare but clinically consequential. SEER data indicate that ANECs carry significantly worse survival compared to pure adenocarcinomas, with hazard ratios exceeding 1.3 119. Due to limited biopsy samples and lack of standardized guidelines, these tumors are often misclassified as conventional adenocarcinomas, missing the chance for neuroendocrine-targeted treatment.

The blurring continues at the interface of mixed and composite tumors. In some cases, neuroendocrine and non-neuroendocrine components coexist in the same lesion—either tightly intermingled or occupying distinct areas. According to current WHO definitions, mixed neuroendocrine-non-neuroendocrine neoplasms (MiNENs) require each component to exceed 30%, but real-world tumors often defy such neat criteria 123-125. Esophageal tumors with small cell and squamous or adenocarcinoma elements, for instance, may resemble MiNENs morphologically, yet fall below formal thresholds. Others show abrupt transitions between components, suggesting collision rather than true cellular biphenotypy. This histologic ambiguity is not academic—clinical outcomes differ. For example, recent analyses suggest that collision-type tumors may demonstrate less aggressive dissemination and better survival than their mixed counterparts 120.

**Figure 5:**
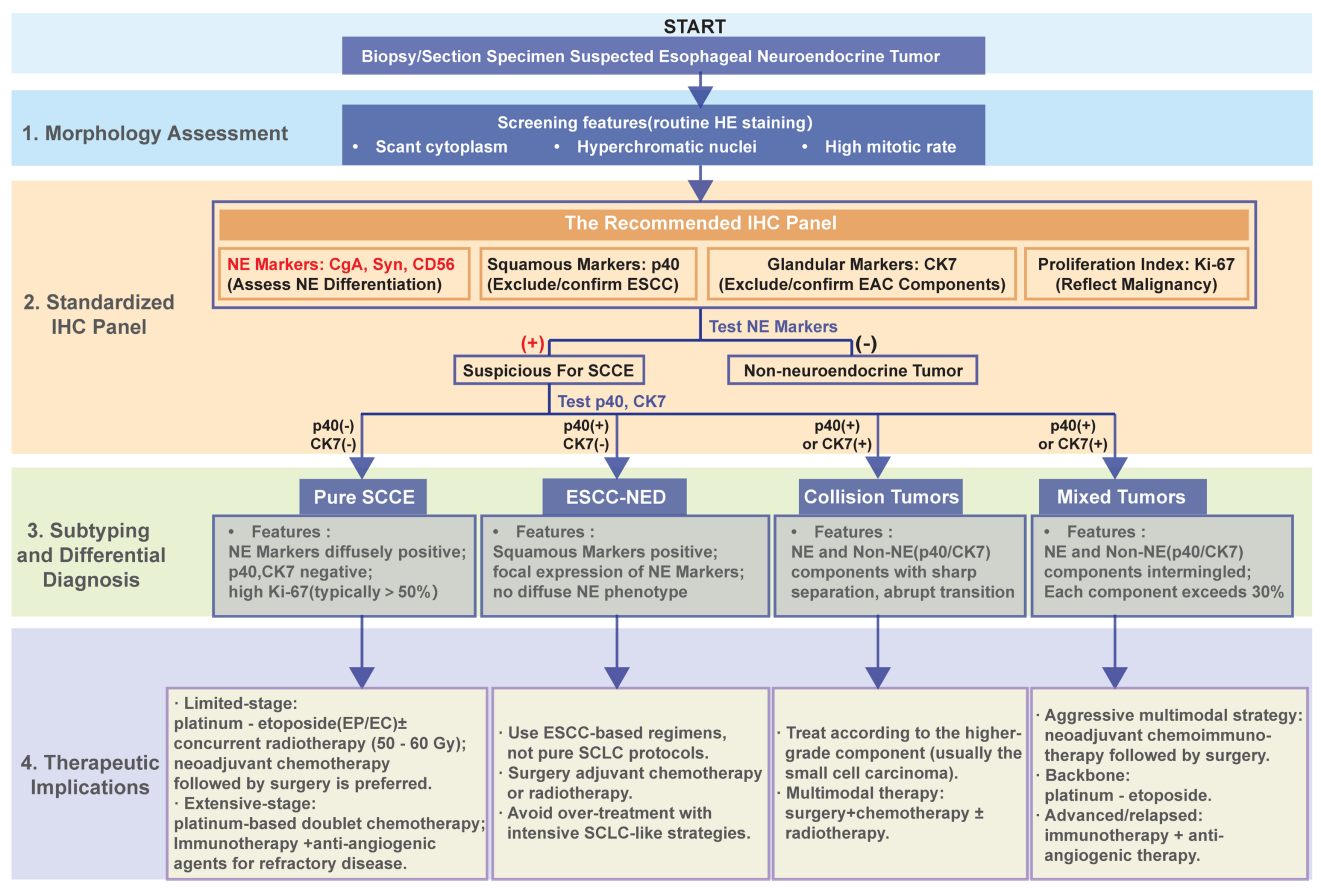
** An IHC-driven diagnostic algorithm for SCCE.** To address blurred pathological boundaries and mitigate the severe consequences of misdiagnosis, this flowchart establishes a standardized four-step workflow: Morphology → IHC → Subtyping → Treatment. Following morphological screening, a recommended IHC panel (CgA, Syn, CD56, p40, CK7, and Ki-67) is employed to definitively differentiate pure SCCE from ESCC-NED, collision, and mixed tumors. By resolving histologic ambiguities, this algorithm prevents inappropriate treatment (e.g., overtreatment with SCLC regimens for ESCC-NED) and ensures accurate, individualized therapeutic strategies. Abbreviations: IHC, immunohistochemistry; SCCE, esophageal small cell carcinoma; HE, hematoxylin and eosin; NE, neuroendocrine; CgA, chromogranin A; Syn, synaptophysin; CD56, cluster of differentiation 56; ESCC, esophageal squamous cell carcinoma; EAC, esophageal adenocarcinoma; CK7, cytokeratin 7; ESCC-NED, esophageal squamous cell carcinoma with neuroendocrine differentiation; EP, etoposide and cisplatin; EC, etoposide and carboplatin; SCLC, small cell lung cancer.

The clinical ramifications of these diagnostic pitfalls are profound. Misidentifying SCCE as conventional ESCC often leads to the omission of essential systemic chemotherapy, potentially resulting in rapid disease progression and early metastasis. Conversely, mistaking ESCC with neuroendocrine-like features for true SCCE may subject patients to the significant toxicities of intensive SCLC-based protocols while depriving them of more appropriate surgical or targeted therapies. Such inaccuracies not only skew prognostic expectations but, more critically, lead to missed therapeutic windows and diminished overall survival. To systematically navigate these ambiguities, Figure 5 outlines an IHC-driven diagnostic algorithm that translates these complex pathological subtypes into distinct therapeutic strategies.

## 5. Discussion

Current clinical evidence confirms that SCCE behaves primarily as a systemic disease. Local control matters but does not determine final survival outcomes. Radiation doses of 45 to 50 Gy are generally sufficient for locoregional management. The true clinical bottleneck remains distant recurrence. Platinum-based chemotherapy serves as the standard anchor for this disease. Regimens like EP or EC yield rapid tumor shrinkage and symptom relief. However, the median overall survival still hovers around 12 months. This reflects a typical pattern of early response followed by early relapse. High proliferative drive coupled with an immunosuppressive niche fuels this rapid initial response. Unfortunately, it also guarantees rapid systemic escape. Overcoming the limited efficacy of standard chemotherapy represents our primary clinical challenge.

For localized stage III disease, the strategic priority must shift. Clinicians should align treatment sequences with underlying tumor biology. Neoadjuvant chemotherapy acts as a vital biologic filter. It tests systemic sensitivity and patient tolerance before any surgical intervention. Clinical observations confirm that patients with pN0 disease and small tumor burdens derive the greatest gain from surgery. Conversely, patients with heavy nodal loads or poor tolerance should avoid upfront resection. For them, completing high-quality concurrent chemoradiotherapy (CCRT) is the more pragmatic path. We hypothesize that future decision models must integrate multiple factors. Tumor length, PET nodal load, and chemotherapy completion probability should guide choices alongside traditional T staging.

**Figure 6:**
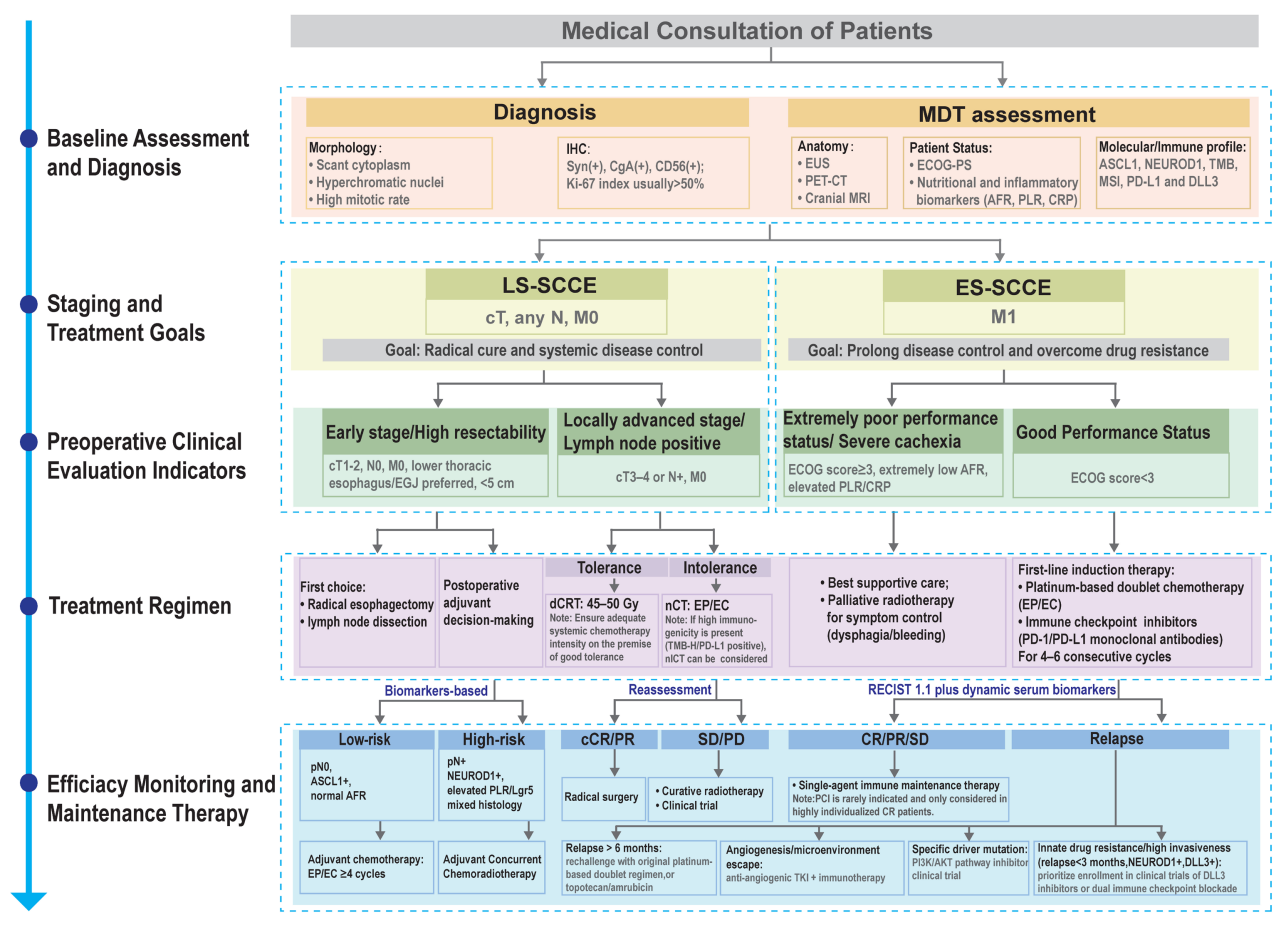
** A structured clinical management roadmap for SCCE.** This framework streamlines therapeutic decision-making by integrating anatomical staging with patient-specific functional and molecular profiles. By synthesizing tumor burden (LS-SCCE vs. ES-SCCE) with performance status and nutritional-inflammatory markers, the algorithm facilitates evidence-based transitions between surgical intervention, definitive chemoradiotherapy, and systemic regimens. For localized disease, it provides a structured logic for navigating the choice between resection and radiotherapy based on nodal load; for advanced or relapsed cases, it delineates the sequence from platinum-based induction to biomarker-driven maintenance and novel targeted salvage. This visualization serves as a pragmatic tool for MDTs to standardize treatment sequencing and individualize patient care. Abbreviations: SCCE, esophageal small cell carcinoma; MDT, multidisciplinary team; EUS, endoscopic ultrasound; PET-CT, positron emission tomography-computed tomography; MRI, magnetic resonance imaging; ECOG-PS, Eastern Cooperative Oncology Group performance status; AFR, albumin-to-fibrinogen ratio; PLR, platelet-to-lymphocyte ratio; CRP, C-reactive protein; TMB, tumor mutational burden; MSI, microsatellite instability; LS-SCCE, limited-stage SCCE; ES-SCCE, extensive-stage SCCE; EGJ, esophagogastric junction; dCRT, definitive chemoradiotherapy; nCT, neoadjuvant chemotherapy; EP, etoposide and cisplatin; EC, etoposide and carboplatin; PCI, prophylactic cranial irradiation; RECIST, Response Evaluation Criteria in Solid Tumors; cCR, clinical complete response; PR, partial response; SD, stable disease; PD, progressive disease; CR, complete response; TKI, tyrosine kinase inhibitor.

In extensive-stage SCCE, overcoming the current treatment ceiling requires precise molecular stratification. Retrospective data show that baseline inflammatory-nutritional indices such as PLR, CRP, and AFR clearly distinguish high- and low-risk groups; BAR (ApoB/ApoA-1) combined with TNM outperforms TNM alone. Pathological markers also offer strong exploratory signals. Tumors enriched with CD8+ and TCF1+ stem-like T cells respond better to chemo- or chemoradiotherapy. Interestingly, a high Ki-67 index often indicates chemosensitivity rather than absolute poor prognosis. Dynamic serum markers like CgA and NSE correlate well with disease control. These predictive tools can transform SCCE from a uniform disease into a carefully stratified condition. Bringing these diverse criteria together, Figure 6 provides a pragmatic clinical roadmap. It is designed to guide multidisciplinary teams (MDT) through complex treatment forks, particularly when navigating surgical tolerance and post-relapse targeted options.

Recent clinical trials provide exploratory evidence for breaking the immunotherapy bottleneck. Anti-angiogenic and immune-based combinations show the first encouraging signals. The phase III NCT05015621 trial demonstrated that sintilimab plus surufatinib markedly improved progression-free survival. Impressively, this combination achieved a 100% Disease control rate (DCR) among patients with NEC after platinum failure. A multicenter study led by Zhang and colleagues provided further support. They reported an objective response rate of 63.6% using surufatinib combined with chemotherapy and sugemalimab in extrapulmonary neuroendocrine carcinoma. Mechanistically, anti-angiogenic therapy starves tumors and normalizes the surrounding vasculature. This process reopens immune access and reverses the immune-desert phenotype of SCCE.

Bispecific antibodies, such as LBL-024, represent a potential therapeutic strategy. By targeting PD-L1 and 4-1BB simultaneously, this drug confines immune stimulation directly to the tumor site. In the 2024 ASCO cohort, LBL-024 monotherapy achieved an objective response rate of 33.3% with a median duration of response of 5.3 months. When combined with platinum and etoposide, the objective response rate boosted to 75%. This included complete responses even in PD-L1-negative patients. Early results suggest that such co-stimulation offers a strong alternative to classical PD-1 monotherapy.

Meanwhile, DLL3 has emerged as a significant molecular target under investigation for neuroendocrine malignancies. It marks neuroendocrine differentiation and shows high expression in approximately 80% of gastrointestinal neuroendocrine carcinomas 126. The CD3 and DLL3 bispecific T-cell engager Tarlatamab has already gained FDA accelerated approval. It showed durable responses in extensive-stage small-cell lung cancer (ES-SCLC). Another agent named BI 764532 further validated this target at the ENETS 2024 conference. It demonstrated objective response rates of 22% and 40% in gastrointestinal and urogenital neuroendocrine carcinomas, respectively. The biological rationale and expression pattern of SCCE closely mirror these diseases. This makes DLL3-directed therapy a highly transferable opportunity. Incorporating DLL3 testing into routine practice is scientifically sound and paves the way for advanced therapies. Whether exploring emerging pan-cancer targets or optimizing classic locoregional and systemic regimens, the clinical management of SCCE is rapidly advancing toward individualized intervention. To visualize this translational landscape, Table 6 summarizes the key clinical trials dedicated to primary SCCE. These studies span multiple intervention phases, ranging from neoadjuvant settings to salvage therapy. In parallel, they prospectively investigate the deep synergy between immune checkpoint blockade and chemoradiation (e.g., the ongoing NCT06478264 trial). These immune combinations and targeted strategies delineate the developmental framework for precision therapy in SCCE.

**Figure 7:**
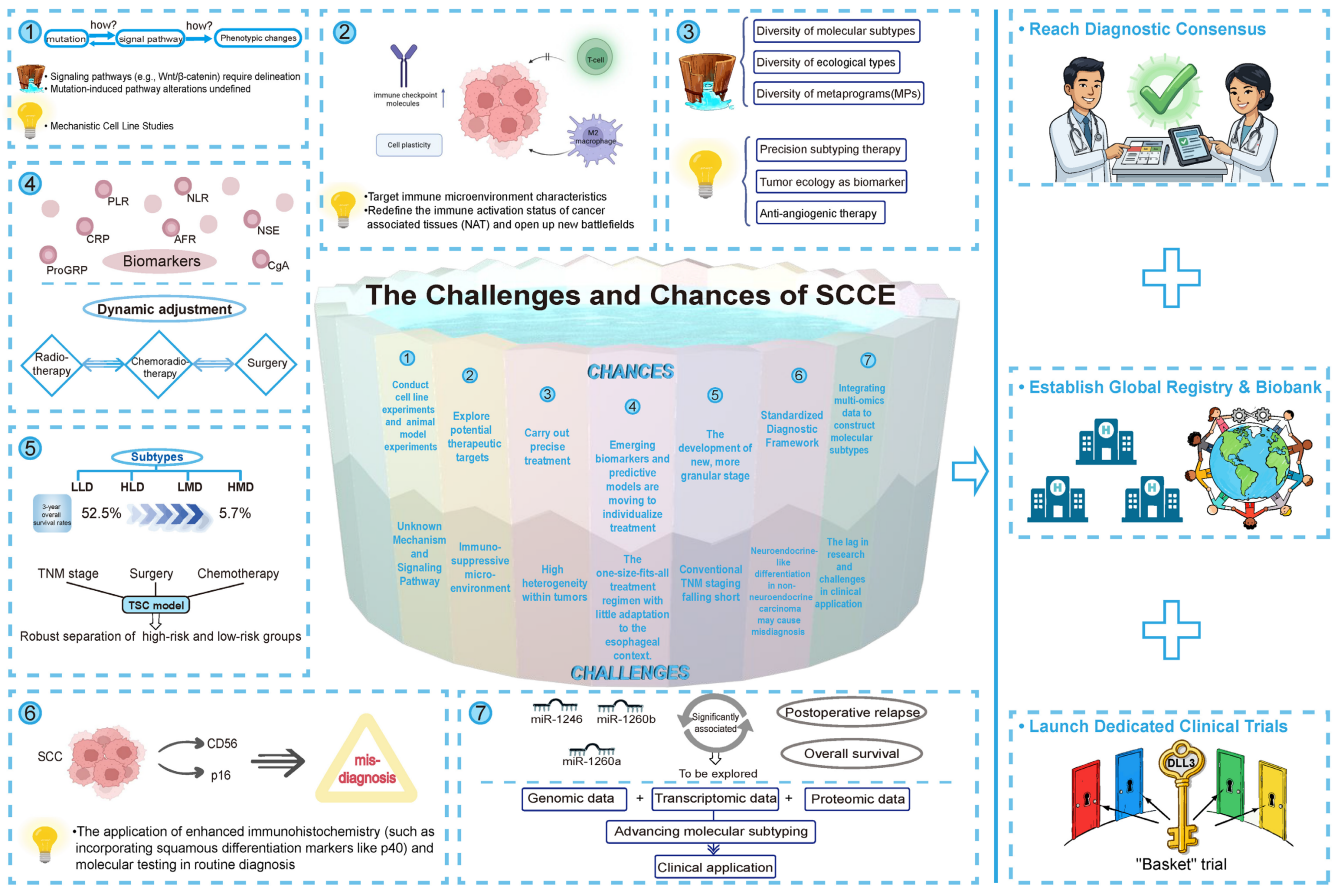
** The “short-barrel effect” in SCCE: linking challenges with opportunities to lift the waterline of progress.** This schematic integrates the dual dimensions of current obstacles and emerging opportunities in SCCE research. Each wooden stave represents a distinct domain—mechanistic insight, model establishment, biomarker discovery, clinical stratification, and diagnostic accuracy—where the height of progress is limited by its weakest link. The lower layer outlines key challenges, including undefined signaling pathways, tumor heterogeneity, diagnostic ambiguity, and fragmented multi-omics data. The upper layer presents corresponding chances: developing representative cell and animal models, exploring therapeutic targets, redefining immune microenvironment features, standardizing diagnostic frameworks, and integrating multi-omics subtyping into clinical application. The right panel outlines the translational ladder—reaching diagnostic consensus, establishing global registries and biobanks, launching dedicated basket trials (e.g., DLL3-directed platforms). Together, this figure embodies the principle that only by repairing the weakest staves—bridging biological understanding with systematic validation—can the overall “water level” of SCCE research and therapy continue to rise. Abbreviations: SCCE, esophageal small cell carcinoma; ProGRP, progastrin-releasing peptide; NLR, neutrophil-to-lymphocyte ratio; NSE, neuron-specific enolase; CRP, C-reactive protein; AFR, albumin-to-fibrinogen ratio; CgA, chromogranin A; PLR, platelet-to-lymphocyte ratio; LLD, low-risk, ASCL1+ subtype; HLD, high-risk, ASCL1+ subtype; LMD, low-risk, mixed neuroendocrine-like subtype; HMD, high-risk, mixed neuroendocrine-like subtype; TNM, tumor-node-metastasis; SCC, squamous cell carcinoma; MPs, metaprograms; miR, microRNA; DLL3, delta-like ligand 3.

Looking forward, SCCE research must evolve from case-driven observations to system-driven evidence. Figure 7 visualizes the evolving roadmap of SCCE research as a wooden barrel and the overall progress of the field depends entirely on repairing its weakest staves. Translating current biological insights into clinical practice requires highly focused strategies. Future efforts should clearly prioritize three core directions. Resolving baseline heterogeneity represents the vital first step. The oncology community urgently needs a standardized pathological diagnostic consensus. Such an agreement will clarify historical ambiguities regarding mixed variants or collision tumors and ensure uniform patient enrollment. With standardized criteria in place, the scale of clinical evidence must improve. Researchers should launch prospective multicenter registry studies. This cooperative approach will effectively break existing data silos. It also provides a robust platform for validating current risk-stratification models across diverse populations. Future therapeutic breakthroughs will rely on innovative trial designs. Investigators must prioritize DLL3-directed basket trials. Evaluating novel agents like bispecific T-cell engager (BiTE) or antibody-drug conjugates (ADCs) within these adaptive frameworks allows direct comparison against standard chemo-immunotherapy regimens.

In short, the priority is clear: a standardized pathological diagnostic consensus, prospective multicenter registry studies, and DLL3 basket trials. What SCCE needs now is not more scattered case reports but stronger prospective collaborations and a new generation of clearly stratified clinical trials. Only through that path can SCCE truly enter the era of precision oncology.

## Figures and Tables

**Table 1:** Summary of Molecular Alterations, Immune Microenvironmental Features, and Potential Therapeutic Targets in SCCE

Mechanism	Detection methods	Potential targets	Corresponding treatment strategies	Study Model	Reference
Gene mutation: p53; Signal Pathway Abnormality: Wnt Pathway; Suppressive Immune Microenvironment:CD276 LAG-3 E2F high expression	RNA isolation and sequencing; Transcriptome Profile	PD-L1; CD276; LAG-3; E2F; p53; Wnt Pathway	ICBT; Targeted inhibition of CD276/B7-H3, LAG-3; Targeting M2-macrophage polarization to improve the immune microenvironment	In vitro	22
Abnormal gene expression: FOXM1& UBE2C upregulation	Differential Expression Analysis; Functional Enrichment Analysis; PPI Network and Hub Gene Screening	FOXM1; UBE2C	CDK1 inhibitors: Rucaparib; EZH2 inhibitors: Tazemetostat (Dabrafenib); Associated with BIRC5: Lapatinib, Paclitaxel; Associated with AURKA: Cisplatin, Sorafenib; Associated with KLF4: Hydroxyurea	In vitro	37
Abnormal gene expression: SOX2 High expression Rb1 Low expression	IHC	SOX2 and Rb1 pathway components	Exploratory stage	In vitro	7
SCCE genomic features: TP53, RB1, NOTCH1, FAT1, FBXW7, PTEN, PAK-1, and other mutations	WES; Targeted Deep Sequencing; CNV microarray	TP53, Rb1, NOTCH1, FAT1, FBXW7, PTEN, PAK-1	Exploratory stage	In vivo	6
Gene mutation: PTEN	Clinical specimens; DNA isolation; EGFR, KRAS and PIK3CA mutation analysis; PTEN mutation analysis	PTEN; PI3K/AKT Pathway; EGFR	EGFR-TKI (in rare mutation-positive cases): Gefitinib	In vitro	36
miRNA regulation:miRNA participates in the initiation and progression of cancer	Perform miRNA array analysis using FFPE tissue samples; Screen for differentially expressed miRNAs and correlate them with survival duration	miRNA	Exploratory stage	In vivo	117
Immune Microenvironment: TME characterized by angiogenesis and immunosuppression; Abnormal gene expression: High MP5 expression	MPs; scRNA-seq; TME subtyping (EC1-5)^1^	EC1 Type Angiogenic Niche; Upregulation of MP5 signature genes	Exploratory stage	In vitro	9

1.EC1-5: Ecotypes 1-5, among which EC1 represents an angiogenic niche with immunosuppressive features. Abbreviations: CNV: Copy Number Variant; FFPE: Formalin-Fixed Paraffin-Embedded; ICBT: Immune Checkpoint Blockade Therapy; IHC: Immunohistochemistry; MPs: Metaprograms; TME: Tumor Microenvironment; WES: Whole Exome Sequencing.

**Table 2:** SCCE Evidence grading and stratification for clinical management

Stratification features in clinical management (staging/nodal status/biomarkers)	Level I	Level II	Level III
Limited-stage, node-negative	Postoperative adjuvant platinum-based chemotherapy, or definitive platinum-based chemoradiotherapy alone 127		
Limited-stage, node-positive	Neoadjuvant platinum-based chemotherapy + surgery 12, or definitive chemoradiotherapy 127	Neoadjuvant PD-1/PD-L1 inhibitor + platinum-based chemotherapy 128	
Extensive-stage, PD-L1 ≥ 1% with high CD8⁺ TIL infiltration	Platinum-based systemic chemotherapy 129	PD-1/PD-L1 inhibitor + platinum-based chemotherapy 81	
Extensive-stage, PD-L1 < 1% with low CD8⁺ TIL infiltration (high DLL3 expression)	Platinum-based systemic chemotherapy 129		DLL3-targeted therapy (e.g., ADCs or bispecific T-cell engagers; clinical trial recommended) [NCT06816394]

Abbreviation: ADCs: Antibody-Drug Conjugates.

**Table 3:** Biomarkers in Esophageal Small Cell Carcinoma: Implications for Precision Oncology

Biomarkers	Biological Attributes and Mechanistic Characteristics	Individualized Clinical Decision-making and Precision Translational Value	Reference
Lgr5	Cancer Stem Cell Marker/Wnt-signaling Cascade Component	High Lgr5 expression correlates with metastasis, advanced stage, chemoresistance, and shortened OS; this independent marker identifies high-risk patients needing early targeted or intensive multidisciplinary therapies.	98
PLR & NLR	Biomarkers of systemic inflammation and immune dysfunction	Pre-treatment stratification and prediction, guiding shifts to combination therapies (e.g. chemo-immunotherapy/anti-angiogenics).	99
AFR	Host Nutritional and Coagulation Imbalance Axis	Reflects cachexia and hypercoagulable states (low AFR/FAR ≥0.08), mandating targeted nutritional prehabilitation and anticoagulant/anti-inflammatory interventions prior to radical esophagectomy or platinum chemotherapy.	102
PARP1	Sensor of DNA Single-Strand Break Repair	Confers synthetic lethality vulnerability, guiding individualized salvage PARPi (e.g., olaparib/rucaparib) therapy in platinum-resistant SCCE.	130
CRP	Biomarker of systemic inflammation	Elevated CRP correlates with advanced T/N stages and shortened OS, serving as a potential surrogate for tumor burden and immune dysregulation.	101
PD-L1	Biomarker of immune cells infiltrating the tumor	High PD-L1 expression predicts prolonged survival and suggests viable immunotherapeutic targets in GI-NEC.	81, 82
CD8⁺ TILs & TCF1⁺CD8⁺	Expression Markers of Tumor Immune Infiltration	CD8⁺ TIL density predicts adjuvant chemotherapy benefit; the TCF1⁺CD8⁺ subset ratio refines prognostic value.Salvageable "adaptive immune tolerance" in SCCE highlights translational potential for immunotherapeutic reversal.	81, 83, 84
TIGIT/CD155 axis	Alternative/Co-inhibitory Immune Checkpoints	High expression correlates with poor prognosis, identifying a potential target to overcome PD-1 resistance. Marker-defined T-cell exhaustion and PD-1 resistance justify clinical enrollment for dual TIGIT/PD-L1 blockade in refractory cohorts.	85, 131
CgA	Neuroendocrine marker	CgA positivity serves as a reliable, favorable prognostic biomarker, facilitating survival stratification across various disease stages.	94, 95

Abbreviations: AFR: Albumin-to-Fibrinogen Ratio; CRP: C-Reactive Protein; GI-NEC: Gastrointestinal poorly differentiated Neuroendocrine Carcinomas; NLR: Neutrophil-Lymphocyte Ratio; OS: Overall Survival.

**Table 4:** Impact of Different Treatment Modalities on Reversing SCCE Resistance and Future Prospects

Treatment Paradigm	Traditional Treatment methods	Therapeutic Advantages	Clinical Bottlenecks/Addressing Deficiencies	Future Directions	Reference
SCLC Regimens (Systemic Chemotherapy)	Etoposide and Cisplatin	Rapid anti-tumor response, quickly alleviating SVCS emergencies; Superior survival benefits for high systemic inflammation (NLR ≥ 3.8) subpopulations	Severe non-hematological toxicity: induces persistent nausea, vomiting, and peripheral neuropathy; Rapid secondary drug resistance: almost inevitable.	Tarlatamab reduces death risk by 40% vs. conventional chemotherapy with higher tumor response rates and more durable responses.	132-137
	Etoposide and Carboplatin	High safety, largely eliminating nephrotoxicity and neurotoxicity without mandatory hydration; Equivalent to cisplatin in OS, PFS, and DCR.	Dose-limiting severe myelosuppression, with high incidence of Grade 3/4 thrombocytopenia and neutropenia; High risk of fatal hemorrhage or febrile neutropenia with infection.		
Surgery (Resection)	Surgery alone in Resectable SCCE	Primary resection rapidly relieves malignant dysphagia (e.g. esophageal obstruction); Sufficient tissue sampling enables precise pTNM staging, the "gold standard" for precision adjuvant therapy.	Fails to prevent early, extensive lymphovascular micrometastasis of SCCE; High surgical trauma risks severe pulmonary infections and anastomotic complications.	Radical resection + neoadjuvant/adjuvant therapy significantly improves survival and prognosis across most stages vs. surgery alone;(e.g. for cT1-2N0M0 SCCE compared with traditional surgery alone, 5 years OS: 32.8% vs. 19.2%)	57, 89, 127
RT	Radiotherapy Alone	Precise non-invasive intervention (esp. IMRT) rapidly alleviates fatal complications (e.g. hemorrhage, tracheal compression); Significantly reduces mortality risk by >40% vs. observation or chemotherapy alone; Irreplaceable salvage value for local tumor ablation in extensive-stage disease with brain/bone metastases.	Fails to prevent hematogenous spread, leading to fatal liver/brain/bone metastases; Rapid early recurrence (67% occur within 12 months post-radiotherapy); High doses risking fatal radiation hemorrhage	MRgRT utilizes high soft-tissue resolution for real-time tracking and respiratory gating; Online adaptation enables real-time plan adjustments for daily tumor and anatomical changes; Tighter volumes minimize cardiopulmonary toxicity.	57, 127, 138
CRT	Etoposide and Cisplatin/Etoposide and Carboplatin+3D-CRT/IMRT	Synergy of CT systemic eradication and RT local ablation; Definite survival benefit: equivalent to radical resection, superior in specific regions.	High toxicity-induced dropout rate; Severe radiation esophagitis causes severe odynophagia, cachexia, and nutritional failure.	Immune synergy: PD-1/PD-L1 inhibitors establish long-term surveillance, prolonging PFS and OS; MRgRT and protectants mitigate cardiopulmonary toxicity and esophagitis, ensuring full-dose/course chemoradiotherapy.	11, 80, 127, 138-141
ICT	PD-1/PD-L1 inhibitors	Avoids cytotoxicity, eliminating severe myelosuppression, alopecia, and gastrointestinal damage; Biomarker-driven long-tail effect: multi-year anti-tumor memory in highly matched minority patients.	Cold tumor: dense stroma excludes T cells. Immune exhaustion: M2, MDSCs, and hypoxia/acidity deplete CD8+ T cells. Monotherapy limit: PD-1 alone fails to breach vascular barriers, risking rapid progression.	Biomarker profiling (PD-L1, CD8+, TMB, ctDNA, TIME) identifies "inflamed tumor" responders; LBL-024 + chemotherapy improves survival (ORR: 66.7%-79.2% across 6-15 mg/kg).	127, 128, 142, 143

Abbreviations: CRT: Chemoradiotherapy; CT: Chemotherapy; DCR: Disease Control Rate; ICT: Immune Checkpoint Therapy; NLR: Neutrophil-Lymphocyte Ratio; ORR: Overall Response Rate; OS: Overall Survival; PFS: Progression-Free Survival; RT: Radiotherapy; SCLC: Small Cell Lung Carcinoma; SVCS: Superior Vena Cava Syndrome; TMB: Tumor Mutational Burden.

**Table 5:** Comparative Analysis of Molecular Subtypes, Immune Microenvironments, and Therapeutic Strategies Across Gastric, Lung, and Esophageal Neuroendocrine Tumors

Primary Site	Subtypes	Characteristics of the Immune Microenvironment	Typical Treatment Strategies	Reference
Gastric	TYPEⅠ^1^	Endocrine markers: CgA (+), NSE, vesicular Monoamine Transporter 2; Expression rate of *MKI67*<2%	Subtotal or total gastrectomy	144-146
	TYPEⅡ^2^	Endocrine markers: CgA (+), NSE, vesicular Monoamine Transporter 2; Expression rate of *MKI67*<2%	Endoscopic resection	
	TYPEⅢ^3^	Endocrine markers: CgA (-); Expression rate of *MKI67*>2%	Subtotal or total gastrectomy associated with lymphadenectomy	
	TYPEⅣ^4^	Endocrine markers: CgA (-), Synaptophysin, NSE, PGP9.5; Expression rate of *MKI67*>30%	/	
Lung	SCLC-A	*ASCL1* and* SLFN11* show significant expression; *TTF1* expression is higher than N-type	High sensitivity to *BCL2* inhibitors	147-149
	SCLC-N	*NEUROD1* show significant expression	High sensitivity to Aurora Kinase inhibitors	
	SCLC-P	*POU2F3* show significant expression; Driven by* POU2F3* gene	High sensitivity to PARPi and antimetabolites	
	SCLC-Y	*YAP1* show significant expression	Targeted therapy: TROP2	
	SCLC-I	High expression of Bruton tyrosine kinase; Massive infiltration of immune cells	Immunotherapy (ICIs); BTK inhibitor	
Esophagus	SCCE-A^5^	Immunosuppressive Microenvironment^6^; High angiogenesis-related markers and signatures PD-L1 in tumor-infiltrating immune cells and CD8+ states	CT+RT; Anti-angiogenesis therapy	9
	SCCE-N^7^	Immunosuppressive Microenvironment; High angiogenesis-related markers and signatures PD-L1 in tumor-infiltrating immune cells and CD8+ states	CT+RT; Anti-angiogenesis therapy	

1. TYPE I: Lesions correspond to the majority of gNETs found in the stomach (70-80%) and they are associated with autoimmune chronic atrophic gastritis (70%-80%) 2. TYPE II: Accompanied by Gastrinoma (7%) 3. TYPE III: Consist of a sporadic lesion and has the greatest potential to generate metastasis 4. TYPE IV: Associated with high malignancy, distinct from Type I/II/III, often poorly differentiated 5. SCCE-A: Enriched in cell-cell adhesion and epidermal differentiation pathways 6. Decreased infiltration of effector CD8+ T cells, an enrichment of activated Treg cells, highly differentiated B and Plasma cells 7. SCCE-N: Enriched in cell cycle, protein metabolism related pathways Abbreviations: CT: Chemotherapy; gNETs: gastric Neuroendocrine Tumors; ICIs: Immune Checkpoint Inhibitors; RT: Radiotherapy.

**Table 6:** Overview of Clinical Trials on Small Cell Carcinoma of the Esophagus

Clinical Trial ID	Primary therapeutic modality	Phase	Potential Mechanism of Action	Target Population
NCT06478264	Adebrelimab Combined With Chemoradiotherapy	Phase II	PD-1/PD-L1 blockade restores T-cell activity. Radiotherapy induces tumor death, neoantigen release (ICD), and PD-L1 upregulation, synergizing to drive the abscopal effect.	Stage T1N0M0: Intolerant to or refusing surgery. Stage T2-4N0-3M0 (AJCC 8th Edition).
NCT03811379	Toripalimab as Monotherapy	Phase II	PD-1 blockade prevents PD-L1/L2 binding, reversing immune tolerance and restoring CTL cytotoxicity.	Patients with advanced or metastatic SCCE refractory to prior chemotherapy.
NCT05173246	Toripalimab Combined With Nab-paclitaxel and Cisplatin or Carboplatin	Phase II	Chemotherapy-induced tumor lysis (via mitotic/DNA damage) releases antigens, synergizing with immune checkpoint blockade (ICB).	Unresectable locally advanced or recurrent/metastatic SCCE.
ChiCTR2300078679	Serplulimab Combined With Paclitaxel And Carboplatin	Phase II	Chemotherapy-induced antigen release synergizes with serplulimab-mediated immune restoration for durable SCCE control.	Patients with locally advanced yet theoretically resectable SCCE.
NCT07155122	Serplulimab Combined With Etoposide and Cisplatin as Neoadjuvant Therapy	Phase II	Chemotherapy-induced ICD enhances antigen exposure, synergizing with serplulimab (anti-PD-1) to amplify anti-tumor immunity.	Treatment-naive patients with potentially resectable, limited-stage primary SCCE.

Abbreviation: ICB: Immune Checkpoint Blockade.

## Abbreviations

3D-CRT: three-dimensional conformal radiotherapy
ADC: antibody-drug conjugate
AFR: albumin-to-fibrinogen ratio
AJCC: American Joint Committee on Cancer
ANEC: adenocarcinoma with neuroendocrine differentiation
ApoA-1: apolipoprotein A-1
ApoB: apolipoprotein B
ASCO: American Society of Clinical Oncology
BAR: ApoB/ApoA-1 ratio
BiTE: bispecific T-cell engager
BTK: Bruton tyrosine kinase
CCRT: concurrent chemoradiotherapy
CgA: chromogranin A
CNV: copy number variant
CR: complete response
CRP: C-reactive protein
CT: computed tomography
CTL: cytotoxic T lymphocyte
DCR: disease control rate
DFS: disease-free survival
dCRT: definitive chemoradiotherapy
EC: etoposide plus carboplatin
EC1: ecotype 1
EMT: epithelial-mesenchymal transition
ENEC: esophageal neuroendocrine carcinoma
EP: etoposide plus cisplatin
ES: extensive stage
ESCC: esophageal squamous cell carcinoma
ESCC-NED: esophageal squamous cell carcinoma with neuroendocrine differentiation
ES-SCLC: extensive-stage small-cell lung cancer
FAR: fibrinogen-to-albumin ratio
FFPE: formalin-fixed paraffin-embedded
GEJ: gastroesophageal junction
GI: gastrointestinal
GI-NEC: gastrointestinal neuroendocrine carcinoma
gNET: gastric neuroendocrine neoplasm
HLD: high-risk localized disease
HMD: high-risk metastatic disease
ICB: immune checkpoint blockade
ICD: immunogenic cell death
ICI: immune checkpoint inhibitor
IHC: immunohistochemistry
IMRT: intensity-modulated radiotherapy
JAK: Janus kinase
LCNEC: large-cell neuroendocrine carcinoma
LLD: low-risk localized disease
LMD: low-risk metastatic disease
LS: limited stage
MDT: multidisciplinary team
MDSC: myeloid-derived suppressor cell
MiNEN: mixed neuroendocrine-non-neuroendocrine neoplasm
miRNA: microRNA
MoA: mechanism of action
MP: metaprogram
MRgRT: magnetic resonance-guided radiotherapy
MSI: microsatellite instability
nCT: neoadjuvant chemotherapy
NCDB: National Cancer Database
NEC: neuroendocrine carcinoma
NED: neuroendocrine differentiation
NGS: next-generation sequencing
nICT: neoadjuvant immunochemotherapy
NLR: neutrophil-to-lymphocyte ratio
NSE: neuron-specific enolase
ORR: objective response rate
OS: overall survival
PARPi: poly(ADP-ribose) polymerase inhibitor
PCI: prophylactic cranial irradiation
PFS: progression-free survival
PLR: platelet-to-lymphocyte ratio
ProGRP: pro-gastrin-releasing peptide
PPI: protein-protein interaction
RT: radiotherapy
SCC: squamous cell carcinoma
SCCE: small cell carcinoma of the esophagus
SCCE-A: ASCL1-dominant SCCE subtype
SCCE-N: NEUROD1-dominant SCCE subtype
scRNA-seq: single-cell RNA sequencing
SCLC: small cell lung cancer
SNRLD: surgery-non-response limited disease
SRD: surgery-responsive disease
SRLD: surgery-responsive limited disease
SVCS: superior vena cava syndrome
Syn: synaptophysin
TIL: tumor-infiltrating lymphocyte
TIME: tumor immune microenvironment
TMB: tumor mutational burden
TMB-H: tumor mutational burden-high
TME: tumor microenvironment
TNM: tumor-node-metastasis
Treg: regulatory T cell
TRM: tissue-resident memory T cell
VASLG: Veterans Administration Lung Study Group
WES: whole-exome sequencing
WHO: World Health Organization
